# Cross-Tissue Transcriptomic Convergence Identifies a Leuko-Cyte-Shared, Myeloid-Enriched Inflammatory Program in Frailty and Osteoarthritis

**DOI:** 10.3390/ijms27156743

**Published:** 2026-07-28

**Authors:** Wang Wei, Bingxiao Pan, Ruiying Li, Dazhi Wang, Zhihao Chen, Zenan Tian, Yisen Feng, Zhikun Jia, Jiajun Jiang, Xiaoyang Wang, Jianlong Ni, Zhibin Shi

**Affiliations:** Department of Sports Medicine and Pediatric Orthopaedics, The Second Affiliated Hospital of Xi’an Jiaotong University, Xi’an Jiaotong University, No. 157, Xiwu Road, Xi’an 710004, China; weiwmed@stu.xjtu.edu.cn (W.W.); bingxiao@stu.xjtu.edu.cn (B.P.); xajdlry@stu.xjtu.edu.cn (R.L.); wdz20000707@stu.xjtu.edu.cn (D.W.); 201004@stu.xjtu.edu.cn (Z.C.); tianzenan@stu.xjtu.edu.cn (Z.T.); fys258456@stu.xjtu.edu.cn (Y.F.); zkjiamed@stu.xjtu.edu.cn (Z.J.); 2192594907@stu.xjtu.edu.cn (J.J.); xywangdoctor@stu.xjtu.edu.cn (X.W.); njl2017@xjtu.edu.cn (J.N.)

**Keywords:** frailty, osteoarthritis, cross-tissue transcriptomics, single-cell RNA sequencing, myeloid inflammation, immune–structural crosstalk

## Abstract

Frailty and osteoarthritis (OA) frequently coexist in older adults, yet the extent to which they share molecular programs across tissues remains unclear. We integrated bulk transcriptomics, peripheral immune and OA meniscal single-cell atlases, inferred cell–cell communication, and an in vitro myeloid–chondrocyte conditioned-medium model. Frailty-associated vastus lateralis and OA synovium shared a 16-gene program enriched for inflammatory and migratory processes, which was refined by protein–protein interaction analysis into a 15-gene hub module (Hub-15). Donor-aware pseudobulk analysis showed coordinated Hub-15 upregulation in CD8 T, natural killer, B, and monocyte/macrophage-like cells, with the highest scores in monocyte/macrophage populations. In the meniscal atlas, donor-level analysis of 7 normal and 6 OA donors identified a 20.5 percentage point lower proportion of inflammatory/stress cells and a 17.3 percentage point higher proportion of fibrochondrocyte-like cells in OA (both false discovery rate = 0.010), whereas state-specific Hub-15 differences were not significant after correction. Exploratory communication analysis nominated directionally consistent candidate myeloid-to-structural signaling axes, although none remained significant after multiple-testing correction. Conditioned medium from cytokine-primed THP-1 cells elicited inflammatory and matrix-catabolic responses in C28/I2 cells. These findings identify a cross-tissue, leukocyte-shared, and myeloid-enriched inflammatory program that converges across independent frailty and OA cohorts, providing a hypothesis-generating framework for future mechanistic testing.

## 1. Introduction

Osteoarthritis (OA) is a leading contributor to pain, disability and reduced quality of life in older adults [1,2]. Contemporary OA biology frames the disease as a whole-joint disorder involving cartilage, synovium, subchondral bone, meniscus and immune-inflammatory remodeling, rather than as a purely mechanical “wear-and-tear” process [3,4]. Inflammatory and senescence-associated mechanisms further contribute to OA onset and progression [5,6]. Additionally, recent evidence indicates that intracellular metabolic dysregulation—such as acetyl-CoA accumulation—can drive chondrocyte senescence and structural cartilage destruction, suggesting that inflammatory and metabolic senescence programs converge during OA progression [7]. Frailty is an aging-associated syndrome characterized by reduced physiological reserve and vulnerability to stressors [8,9,10]. Clinical studies have linked OA with greater frailty burden and poorer physical outcomes, and frailty has been associated with worsening knee pain trajectories [11,12,13]. Cross-sectional studies in community-dwelling older adults and meta-analyses demonstrate a significantly higher prevalence of sarcopenia or frailty among individuals with knee OA, further highlighting the intricate intersection among frailty, muscle dysfunction, and OA [14,15].

Chronic low-grade inflammation provides a plausible biological context for this overlap. In OA, synovial activation is observed across disease stages and is associated with progression-related inflammatory remodeling [16,17]. Synovial macrophages and macrophage-derived mediators contribute to inflammatory and matrix-destructive responses [18,19], while pro-inflammatory cytokines and macrophage polarization shape cartilage catabolic programs [20,21,22]. In frailty and aging, inflammaging and chronic inflammatory tone are well-recognized features [23,24,25], and altered monocyte and dendritic-cell phenotypes have been reported [26,27]. The identification of a shared transcriptomic program between frailty-associated vastus lateralis muscle and OA synovium raises the possibility that common systemic inflammatory processes may contribute to both tissue contexts. Altered circulating myeloid phenotypes represent one hypothesis-generating cellular link, although the present unmatched datasets do not directly test cellular trafficking or temporal continuity. We hypothesized that persistent inflammatory conditioning associated with frailty could prime circulating monocytes toward a heightened inflammatory state, potentially contributing to synovial or meniscal remodeling after tissue recruitment. Within this hypothesis-generating systemic-to-local framework, circulating monocytes represent a plausible mobile conduit, whereas recruited and tissue-resident macrophages may contribute to the local expression of the shared inflammatory program.

Bulk transcriptomics can identify shared genes and pathways across frailty-associated skeletal muscle and OA tissue datasets, but it cannot define the cell states in which those programs operate. Single-cell RNA sequencing can localize shared programs in OA tissue and immune compartments [28,29]. The present single-cell analyses were anchored in GSE157007 and GSE220243 [30,31] and used established Seurat/Harmony integration [32,33], SingleR-assisted annotation [34] and precomputed marker-defined state-ordering analyses interpreted within established pseudotime principles [35]. Inferred cell–cell communication methods can nominate candidate sender–receiver relationships between myeloid cells and tissue-resident structural states [36].

Here, we integrated bulk transcriptomic datasets, peripheral immune and OA knee-tissue single-cell atlases, inferred myeloid-to-structural communication analysis and an in vitro THP-1/C28/I2 conditioned-medium model. We conceptualized the protein–protein interaction (PPI)-derived 15-gene hub inflammatory/migration module (Hub-15) as a leukocyte-shared inflammatory program with myeloid-enriched single-cell localization and association with remodeling-related meniscal structural states, while communication analysis captured a complementary layer of candidate paracrine outputs. We then tested whether a cytokine-primed myeloid milieu was biologically competent to induce OA-relevant inflammatory and catabolic responses in chondrocyte-like cells.

## 2. Results

### 2.1. Dataset-Specific Transcriptional Landscapes in Frailty-Associated Vastus Lateralis and OA Synovium

**Figure 1:**
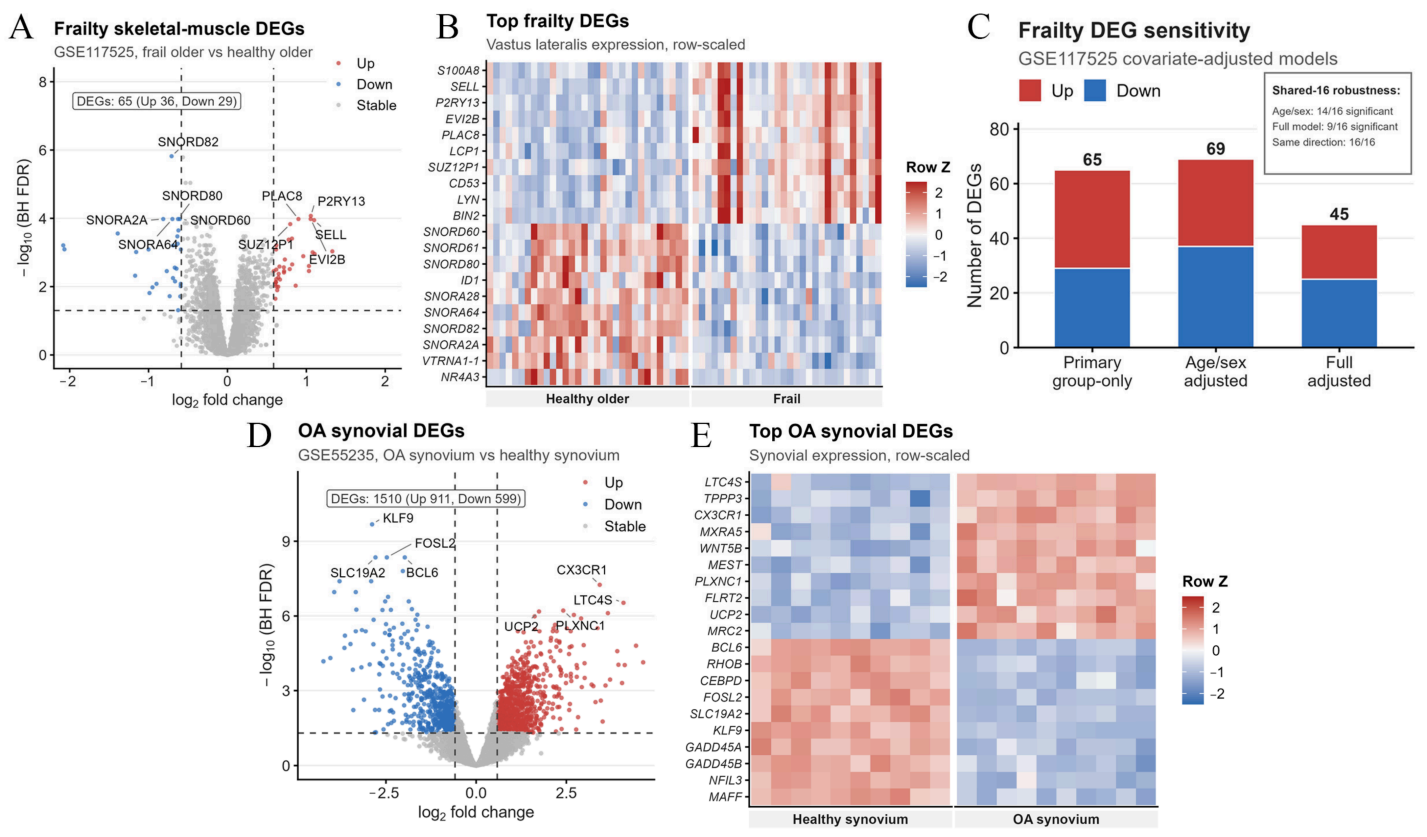
Dataset-specific bulk transcriptomic landscapes and frailty-model sensitivity. (**A**) Volcano plot for frail versus healthy older baseline vastus lateralis samples in GSE117525 (healthy older *n* = 32; frail older *n* = 30). DEGs were defined by BH FDR < 0.05 and |log2FC| > log2(1.5). (**B**) Row-scaled heatmap of top frailty-associated DEGs. (**C**) Stacked DEG counts for the primary group-only, age/sex-adjusted and fully adjusted frailty models. The inset summarizes the robustness of the 16 genes later shared with OA: 14/16 remained significant after age/sex adjustment, 9/16 after full adjustment and 16/16 retained direction across frailty models. (**D**) Volcano plot for OA versus healthy synovium in GSE55235 (*n* = 10 per group) using the same DEG threshold. (**E**) Row-scaled heatmap of top OA synovial DEGs. Model overlap, threshold sensitivity and cohort quality-control analyses are shown in Supplementary Figure S1. Abbreviations: BH, Benjamini–Hochberg; DEG, differentially expressed gene; FDR, false discovery rate; log2FC, log2 fold change; OA, osteoarthritis.

To establish the transcriptional foundations of cross-tissue convergence, we first defined the dataset-specific expression landscapes of frailty-associated vastus lateralis and OA synovium. GSE117525 was analyzed as a frailty-associated skeletal muscle dataset, retaining baseline untrained older vastus lateralis samples only. The primary limma model compared frail older adults with healthy older controls (*n* = 30 versus *n* = 32), yielding 65 differentially expressed genes (DEGs) at a Benjamini–Hochberg-adjusted false discovery rate (BH FDR) < 0.05 and an absolute log2 fold change (|log2FC|) > log2(1.5), including 36 upregulated and 29 downregulated genes (Figure 1A,B). Covariate-adjusted sensitivity models yielded 69 DEGs after age/sex adjustment and 45 DEGs after fuller adjustment for age, sex, body mass index and supplement status (Figure 1C; Figure S1). Importantly, the 16 genes subsequently shared with OA showed striking robustness to clinical adjustment: 14 remained significant after age/sex adjustment, 9 retained strict significance in the fully adjusted model and all 16 preserved their effect direction across models. This complete directional concordance indicates that the shared trajectory was not driven by a reversal under demographic or clinical covariate control. In GSE55235, OA synovium was compared with healthy control synovium (*n* = 10 versus *n* = 10) using log2-transformed gene-level expression, identifying 1510 OA-associated DEGs, including 911 upregulated and 599 downregulated genes (Figure 1D,E). Supplementary quality-control panels summarize cohort composition, retained expression features, threshold sensitivity and model-overlap behavior (Figure S1). Complete primary, adjusted, stability and threshold-sensitivity outputs are provided in Table S2A–F and Source Data Figure 1.

### 2.2. A Shared Frailty–OA Differential-Expression Program Is Enriched for Inflammatory and Chemotactic Pathways

**Figure 2:**
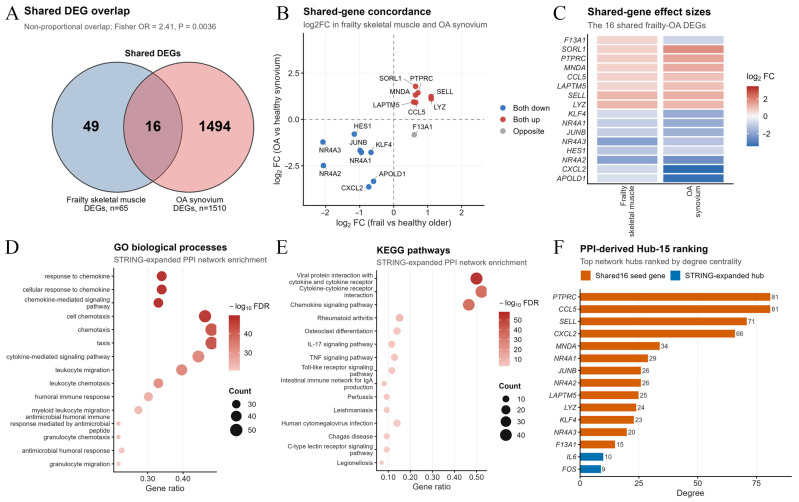
Shared frailty-OA DEGs, interaction-network enrichment and the PPI-derived Hub-15 module. (**A**) Overlap between 65 frailty skeletal muscle DEGs and 1510 OA synovial DEGs, identifying 16 shared genes (one-sided Fisher exact/hypergeometric odds ratio = 2.41; *p* = 0.0036). The circle area is illustrative and not proportional to set size. (**B**) Cross-dataset concordance of shared-gene log2 fold changes; red and blue denote concordant up- and downregulation, respectively, and gray denotes opposite direction. (**C**) Two-column heatmap of shared-gene effect sizes. (**D**,**E**) GO biological process and KEGG enrichment of the STRING-expanded shared-gene interaction neighborhood. (**F**) Degree ranking of the final Hub-15; orange denotes genes from the shared-16 set, and blue denotes STRING-added hubs *IL6* and *FOS*. Supplementary Figure S2 separates strict shared-16 and Hub-15 GO enrichment from expanded-network KEGG and displays centrality robustness and module composition. Abbreviations: DEG, differentially expressed gene; GO, Gene Ontology; Hub-15, PPI-derived 15-gene hub inflammatory/migration module; KEGG, Kyoto Encyclopedia of Genes and Genomes; OA, osteoarthritis; PPI, protein–protein interaction; STRING, Search Tool for the Retrieval of Interacting Genes/Proteins.

To identify a shared molecular denominator between systemic frailty-associated muscle perturbation and local joint pathology, we evaluated the transcriptomic intersection of the two bulk datasets. The overlap analysis identified 16 shared genes: *SELL*, *NR4A3*, *NR4A2*, *HES1*, *LYZ*, *PTPRC*, *KLF4*, *LAPTM5*, *CCL5*, *SORL1*, *APOLD1*, *F13A1*, *MNDA*, *NR4A1*, *JUNB* and *CXCL2* (Figure 2A). A one-sided Fisher exact/hypergeometric test against the harmonized common measured-gene universe indicated that the overlap exceeded random expectation (odds ratio = 2.41; *p* = 0.0036). The overlap comprised 16 shared genes, 49 frailty-only genes and 1494 OA-only genes; the complete 2 × 2 table and universe definition are provided in Source Data. Fifteen of the 16 shared genes were directionally concordant across the two contrasts, spanning concordantly upregulated genes such as *SORL1*, *PTPRC*, *SELL*, *LYZ*, *MNDA*, *LAPTM5* and *CCL5* as well as concordantly downregulated genes such as *NR4A2*, *NR4A3*, *HES1*, *JUNB*, *KLF4*, *NR4A1*, *APOLD1* and *CXCL2*. *F13A1* showed opposite-direction effects (Figure 2B,C). Gene-level overlap, contingency-table and directionality outputs are provided in Table S3A–E and Source Data Figure 2.

Functional analyses were separated by gene-set scope. Strict Gene Ontology (GO) analyses of the 16 shared genes and of the final Hub-15 module supported leukocyte adhesion, cytokine response, immune effector and related biological processes (Figure S2A,B). The main GO and Kyoto Encyclopedia of Genes and Genomes (KEGG) panels used an interaction neighborhood expanded with the Search Tool for the Retrieval of Interacting Genes/Proteins (STRING) because the displayed term counts exceeded the input sizes of the strict gene sets. This expanded network highlighted chemokine responses, cell chemotaxis, leukocyte migration, cytokine-cytokine receptor interaction, chemokine signaling, rheumatoid arthritis, osteoclast differentiation, interleukin-17 (IL-17), tumor necrosis factor (TNF) and Toll-like receptor pathways (Figure 2D,E; Figure S2C). Network centrality analyses prioritized a 15-gene hub inflammatory/migration module consisting of *PTPRC*, *CCL5*, *SELL*, *CXCL2*, *MNDA*, *NR4A1*, *NR4A2*, *JUNB*, *LAPTM5*, *LYZ*, *KLF4*, *NR4A3*, *F13A1*, *IL6* and *FOS* (Figure 2F; Figure S2D–F). The module retained 13 of the 16 shared genes and added *IL6* and *FOS* as STRING-derived hubs; *HES1*, *SORL1* and *APOLD1* were not retained in the top-15 module. This PPI-derived Hub-15, rather than the complete shared-16 set, was used for primary downstream single-cell scoring. Scope-specific enrichment results, STRING nodes and edges, centrality rankings and Hub-15 membership are provided in Table S4A–H and Supplementary Notes S3 and S4.

To address the potential confounding effect of immune-cell infiltration, we performed a tissue-deconvolution sensitivity analysis, which revealed elevated immune-lineage scores in both frail skeletal muscle and OA synovium. However, following adjustment for the composite Microenvironment Cell Populations-counter (MCP-counter) immune score, the directional shared-16 effect remained highly significant in GSE117525, despite its magnitude being attenuated by 36.2% (adjusted β = 0.546, 95% CI: 0.272–0.820; *p* = 1.86 × 10^−4^). In GSE55235, the shared-16 effect demonstrated a negligible attenuation of 2.3% and remained highly significant (adjusted β = 1.282, 95% CI: 0.841–1.724; *p* = 1.12 × 10^−5^). Remarkably, all 16 shared genes maintained their within-dataset effect directions after immune adjustment, and the adjusted cross-tissue gene-effect concordance remained robust (Table S11 and Supplementary Note S8). Collectively, these results indicate that while local immune-cell abundance contributes to the bulk shared-signature signal, it does not exclusively drive the observed cross-tissue transcriptomic convergence.

### 2.3. Peripheral Single-Cell Analysis Identifies a Leukocyte-Shared, Myeloid-Enriched Hub-15 Program

**Figure 3:**
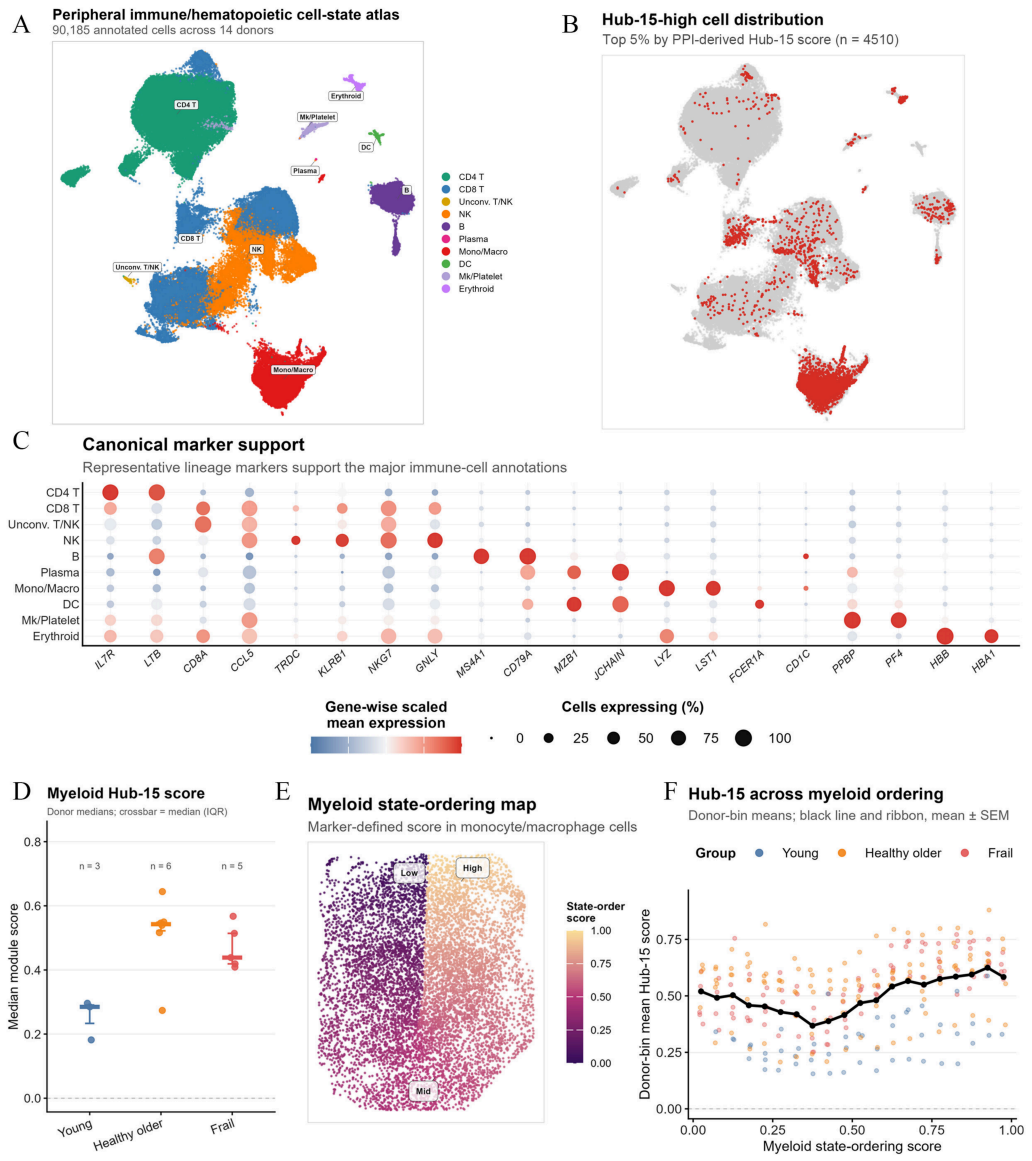
Immune single-cell localization and myeloid-state organization of the PPI-derived Hub-15 module. (**A**) UMAP atlas of 90,185 major-class-annotated immune and hematopoietic cells from 14 GSE157007 donors. (**B**) The top 5% of cells by the annotated-atlas Hub-15 score distribution (*n* = 4510) highlighted in red over the complete gray atlas. (**C**) Canonical-marker dot plot supporting the ten displayed major immune/hematopoietic class annotations. (**D**) Donor-level median Hub-15 scores in monocyte/macrophage-like cells across Young, Healthy older and Frail groups; each point represents one donor, and the crossbar shows the median and interquartile range. (**E**) Marker-defined myeloid transcriptional state-ordering map, interpreted as state organization rather than temporal lineage progression. (**F**) Hub-15 activity across the myeloid ordering score; colored points denote donor-bin means, and the black line and ribbon denote mean ± SEM. Formal Frail versus Healthy older class-specific inference used donor × cell-class pseudobulks and is shown in Supplementary Figure S3. Abbreviations: DC, dendritic cell; Mk, megakaryocyte; IQR, interquartile range; Hub-15, PPI-derived 15-gene hub inflammatory/migration module; NK, natural killer; PPI, protein–protein interaction; SEM, standard error of the mean; UMAP, uniform manifold approximation and projection.

To deconvolve the cellular origins of this frailty-OA convergent program within the immune compartment, we mapped Hub-15 across the updated quality-control GSE157007 peripheral single-cell object used for donor-aware inference. The quality-controlled object contained 91,538 cells from 14 donors. Of these, 90,185 cells received one of the ten displayed major immune/hematopoietic class annotations and were used for the atlas, top-5% and canonical-marker visualizations: 23,822 Young, 47,444 Healthy older and 18,919 Frail cells. The displayed classes were CD4 T, CD8 T, natural killer (NK), unconventional T/NK, B, plasma, monocyte/macrophage-like, dendritic, megakaryocyte/platelet and erythroid cells. The top 5% of the annotated atlas by Hub-15 score (*n* = 4510 cells) were distributed non-uniformly, with the strongest concentration in monocyte/macrophage regions and smaller high-scoring subsets in lymphoid and other immune compartments (Figure 3A–C; Figure S3A–D). Cell-count summaries, annotation mapping and Hub-15 gene-detection records are provided in Table S5A–C.

Within monocyte/macrophage-like cells, donor-median Hub-15 scores were lower in young donors and elevated in both older groups, with overlap between Healthy older and Frail donors. The marker-defined myeloid state-ordering map and donor-binned trends were used descriptively to visualize transcriptional organization rather than temporal progression (Figure 3D–F). For formal Frail versus Healthy older inference, raw unique molecular identifier (UMI) counts were summed within donor × major-cell-class pseudobulks, requiring at least 20 cells per donor-class unit and at least three donors per group. Six of 12 configured classes met these criteria. A limma-voom fry gene-set test, with BH correction across evaluable classes, identified coordinated Hub-15 upregulation in CD8 T cells (BH FDR = 0.0268), NK cells (BH FDR = 0.0244), B cells (BH FDR = 0.0244) and monocyte/macrophage-like cells (BH FDR = 0.0244), whereas CD4 T and megakaryocyte/platelet cells were not significant (Figure S3E). Unconventional T/NK, plasma, dendritic, erythroid, cycling and hematopoietic stem and progenitor cell (HSPC)-like classes lacked sufficient donor coverage. Gene-level edgeR analysis identified 4, 4, 2 and 5 Hub-15 genes with FDR < 0.05 in CD8 T, NK, B and monocyte/macrophage-like cells, respectively (Figure S3G). The coordinated effects across three lymphoid classes and one myeloid class indicate a leukocyte-shared response to frailty, whereas the concentration of the highest absolute single-cell scores in monocyte/macrophage regions supports myeloid enrichment rather than myeloid specificity. Summary module-score, composition and state-ordering outputs are provided in Table S5D–F; complete pseudobulk module-level, gene-level and donor-coverage outputs are provided in Source Data Figure 3.

### 2.4. OA Meniscal Remodeling Places Hub-15 Within State-Specific Structural Programs

**Figure 4:**
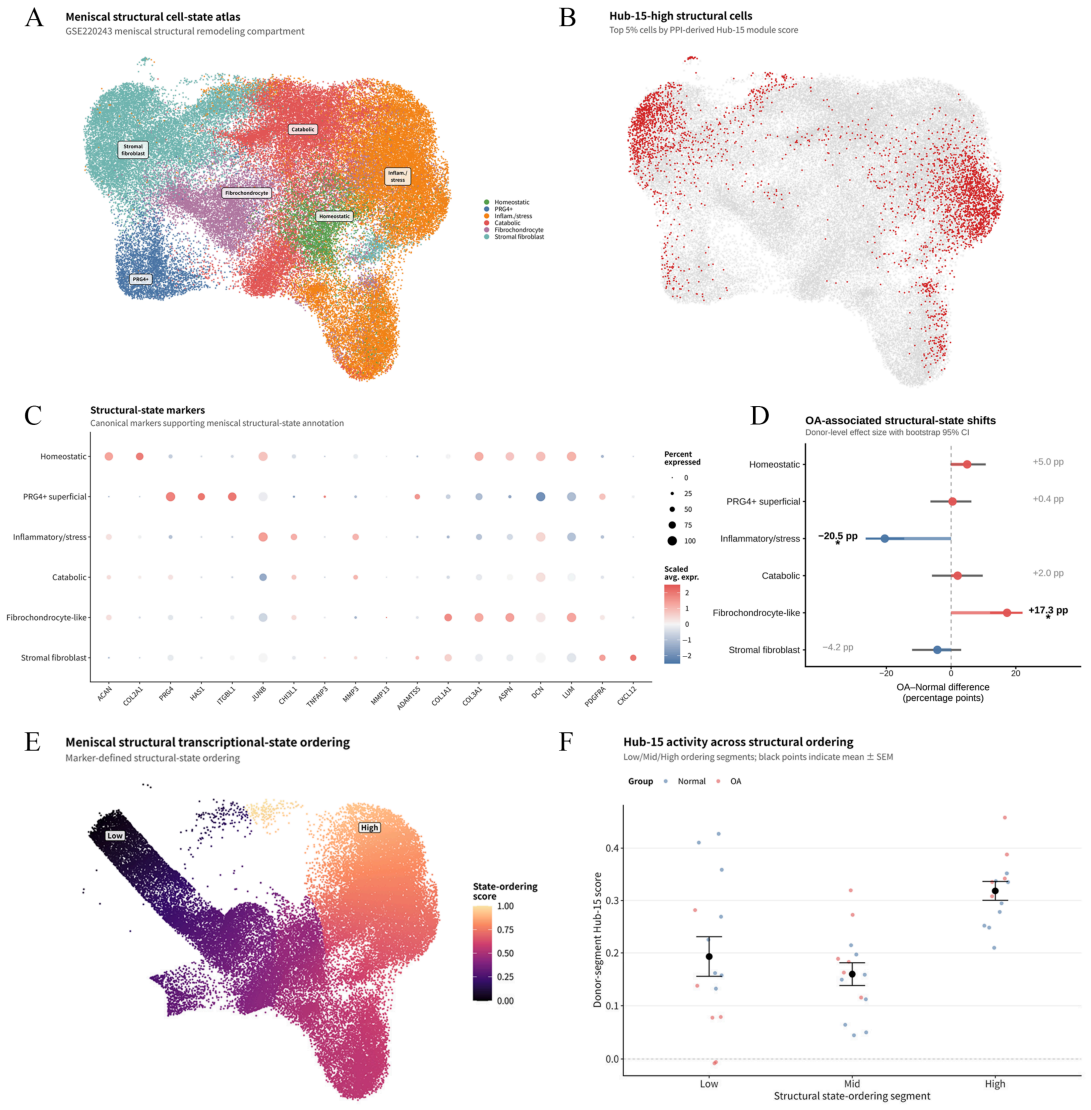
OA-associated meniscal structural-state redistribution and Hub-15 organization. (**A**) UMAP atlas of 71,196 meniscal structural cells from 14 regional samples representing 13 donors (8 Normal samples from 7 donors and 6 OA samples from 6 donors). GSM6797160 and GSM6797161 are avascular and vascular regions from the same Normal Donor1. (**B**) Cells in the top 5% of the structural-atlas Hub-15 score distribution highlighted in red. (**C**) Canonical-marker dot plot supporting structural-state annotations. (**D**) Donor-level OA-Normal differences in structural-state fractions after pooling the two Normal Donor1 regions; inflammatory/stress cells were reduced by 20.5 percentage points and fibrochondrocyte-like cells increased by 17.3 percentage points in OA, both BH FDR = 0.0102. Error bars show 2000-replicate bootstrap 95% confidence intervals. * BH-adjusted *p* < 0.05. (**E**) Marker-defined structural state-ordering map, interpreted as transcriptional organization rather than temporal progression. (**F**) Descriptive donor-segment Hub-15 scores across Low, Mid and High ordering segments; black points and error bars denote mean ± SEM. Abbreviations: BH, Benjamini–Hochberg; FDR, false discovery rate; Hub-15, PPI-derived 15-gene hub inflammatory/migration module; OA, osteoarthritis; SEM, standard error of the mean; UMAP, uniform manifold approximation and projection.

To determine how the convergent inflammatory program is embedded within local joint remodeling, we mapped Hub-15 across OA meniscal structural-cell states in GSE220243. The structural atlas contained 71,196 cells from 14 regional meniscal samples representing 13 independent donors: 42,779 cells from 8 Normal samples derived from 7 donors and 28,417 cells from 6 OA samples derived from 6 donors. GSM6797160 and GSM6797161 represented avascular and vascular regions, respectively, from the same Normal Donor1. Atlas-level visualization retained all 14 regional samples, whereas inferential analyses were specified at the independent-donor level, with the two Normal Donor1 regions pooled before calculation of donor-level summaries. Six structural states were resolved: homeostatic, PRG4+ superficial, inflammatory/stress, catabolic, fibrochondrocyte-like and stromal fibroblast (Figure 4A; Figure S4A–C). Hub-15-high cells occupied discrete remodeling-associated regions, particularly inflammatory/stress and stromal/fibroblast compartments (Figure 4B,C).

After pooling the two Normal Donor1 regions, donor-level structural-state fractions were compared between 7 Normal and 6 OA donors. OA showed a 20.5 percentage point lower inflammatory/stress fraction, with a bootstrap 95% confidence interval (CI) of −26.4 to −14.5 percentage points (BH FDR = 0.0102) and a 17.3 percentage point higher fibrochondrocyte-like fraction (95% CI, 12.1 to 22.1 percentage points; BH FDR = 0.0102) (Figure 4D; Figure S4D). Homeostatic, PRG4+ superficial, catabolic and stromal fibroblast fractions did not differ after BH correction. State-specific donor-median Hub-15 scores showed directional differences, including lower values in PRG4+ superficial and fibrochondrocyte-like states and higher values in inflammatory/stress cells, but none of the six states reached BH FDR < 0.05 (Figure S4E,F). Along the marker-defined structural ordering, Hub-15 activity was highest in the High segment (mean 0.320) relative to the Low (0.183) and Mid (0.158) segments; these segment summaries were descriptive and do not imply temporal lineage progression (Figure 4E,F; Figure S4G,H). Together, these results distinguish a robust OA-associated redistribution of structural states from more heterogeneous within-state Hub-15 score differences. Complete structural-cell counts, marker-detection audits, threshold-sensitivity analyses, donor-corrected structural-state fractions, state-specific Hub-15 effects, and ordering/module-dynamics summaries are provided in Table S6A–H. As a sensitivity analysis, the original shared-16 signature yielded cell-class and structural-state effect estimates closely concordant with those obtained using Hub-15 in GSE157007 and GSE220243 (Pearson r = 0.993 and 0.951, respectively). Positive Frail-versus-Healthy older module effects in CD8 T, NK, and monocyte/macrophage-like cells and the relative prominence of the inflammatory/stress structural state were retained, although the B-cell result remained directionally consistent but no longer passed BH correction. These findings indicate that the principal downstream patterns were not dependent on the STRING-added IL6 and FOS genes (Table S10 and Supplementary Note S7).

### 2.5. Exploratory CellChat Analysis Nominates Candidate OA Myeloid-to-Structural Communication Axes

**Figure 5:**
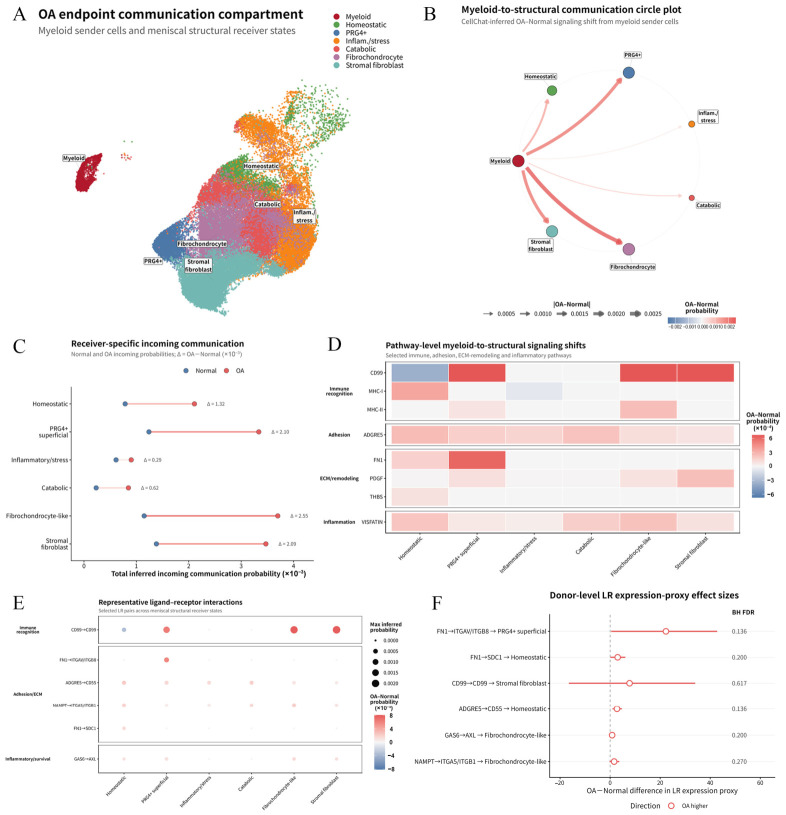
Exploratory OA myeloid-to-meniscal structural communication potential. (**A**) UMAP framework containing myeloid sender cells and six meniscal structural receiver states. (**B**) Directed circular network summarizing OA-Normal shifts in inferred myeloid-to-structural communication potential; edge width and color encode the magnitude and direction of the inferred difference. (**C**) Receiver-specific incoming communication potential in Normal and OA groups. (**D**) Selected pathway-level shifts across receiver states. (**E**) Representative ligand–receptor interactions, with point size encoding maximum inferred probability and color encoding OA-Normal difference. (**F**) Independent-donor expression-proxy effect-size forest after pooling GSM6797160 and GSM6797161 as Normal Donor1. Points denote OA-Normal mean differences, horizontal lines denote 2000-replicate bootstrap 95% confidence intervals and right-side values denote BH FDR after correction across six axes. All six effects were OA-directed, but none reached BH FDR < 0.05. Four axes used 7 Normal versus 6 OA donors; the two homeostatic-receiver axes used 6 versus 6 donors because one Normal donor did not meet the prespecified receiver-cell threshold. CellChat and expression-proxy results are exploratory, computational, expression-based inferences and do not establish ligand secretion, physical binding, receptor activation, or causality. Abbreviations: BH, Benjamini–Hochberg; FDR, false discovery rate; OA, osteoarthritis; UMAP, uniform manifold approximation and projection; ECM, extracellular matrix; LR, ligand–receptor; MHC-I, major histocompatibility complex class I; MHC-II, major histocompatibility complex class II; PDGF, platelet-derived growth factor; THBS, thrombospondin.

To explore whether the leukocyte-shared, myeloid-enriched Hub-15 state was accompanied by candidate changes in paracrine communication, we performed an exploratory CellChat analysis of myeloid-to-meniscal structural communication in Normal and OA tissues. The communication compartment included 73,271 cells from the same 14 regional samples representing 13 donors, comprising 43,247 cells from 8 Normal regional samples (7 donors) and 30,024 cells from 6 OA samples (6 donors). CellChat networks were inferred at the disease-group level using all retained cells, and the two regions from Normal Donor1 were not interpreted as independent donors in any downstream inferential support analysis. OA showed broader inferred outgoing myeloid communication toward multiple structural receiver states, including PRG4+ superficial, fibrochondrocyte-like, stromal fibroblast and homeostatic compartments (Figure 5A–C; Figure S5A–D).

Exploratory pathway-level summaries highlighted candidate immune-recognition, adhesion, extracellular matrix (ECM)/remodeling, and inflammatory/survival programs, including CD99, FN1, major histocompatibility complex class I (MHC-I), ADGRE5, major histocompatibility complex class II (MHC-II), platelet-derived growth factor (PDGF), VISFATIN, and thrombospondin (THBS) signaling (Figure 5D; Figure S5E). Representative ligand–receptor maps nominated FN1-ITGAV/ITGB8, FN1-SDC1, CD99-CD99, ADGRE5-CD55, GAS6-AXL and NAMPT-ITGA5/ITGB1 as candidate axes (Figure 5E; Figure S5F). Donor-level expression-proxy support was recalculated using independent donors, with GSM6797160 and GSM6797161 pooled as Normal Donor1 before estimation. All six prioritized axes exhibited positive OA-Normal effects. The largest effect was observed for FN1-ITGAV/ITGB8 toward PRG4+ superficial cells (mean difference, 22.30; bootstrap 95% CI, 0.45 to 42.79; nominal *p* = 0.0383; BH FDR = 0.136). ADGRE5-CD55 toward homeostatic cells also showed a positive effect (mean difference, 2.75; 95% CI, 0.97 to 4.69; nominal *p* = 0.0453; BH FDR = 0.136). However, none of the six axes retained statistical significance after rigorous BH correction across the comparisons (Figure 5F; Figure S5G; Table S7H–K; Source Data Figure 5). Given the limited donor-level sample size (7 Normal versus 6 OA, with 6 versus 6 evaluable donors for the two homeostatic-receiver axes), these results are conservatively interpreted as directionally coherent, transcriptomically supported and hypothesis-generating candidates requiring functional corroboration.

Together, Hub-15 localization and the exploratory CellChat analysis provide complementary descriptive layers. Hub-15 defines a leukocyte-shared inflammatory state with myeloid-enriched localization, whereas CellChat nominates candidate paracrine outputs associated with that state. These communication outputs are interpreted as hypothesis-generating candidates rather than validated ligand–receptor interactions or established signaling mechanisms.

### 2.6. A Primed Myeloid-Conditioned Milieu Elicits OA-Relevant Responses in C28/I2 Cells

**Figure 6:**
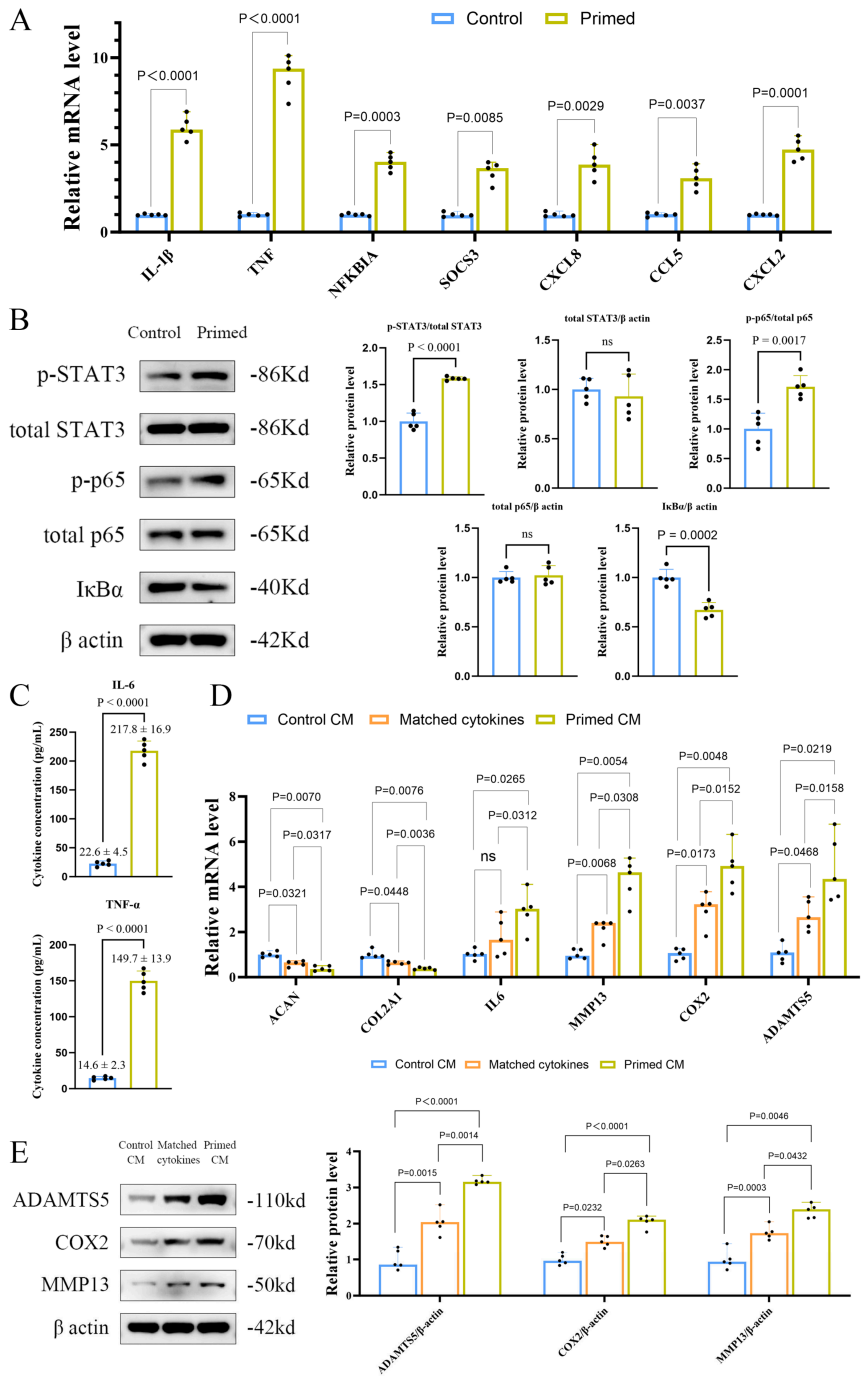
Primed THP-1 conditioned medium induces inflammatory and matrix-catabolic responses in C28/I2 cells that are not fully reproduced by matched TNF-α and IL-6. (**A**) RT-qPCR analysis of IL1B, TNF, NFKBIA, SOCS3, CXCL8, CCL5, and CXCL2 in paired control and cytokine-primed THP-1 cells. (**B**) Representative western blots and densitometric quantification of STAT3- and NF-κB-associated signaling. Phosphorylated STAT3 was normalized to total STAT3, and phosphorylated p65 was normalized to total p65. Total STAT3, total p65, and IκBα were normalized to β-actin. (**C**) ELISA quantification of IL-6 and TNF-α in Control-CM and Primed-CM collected after priming, PBS washing, medium replacement, and 24 h of conditioning. Concentrations shown represent the undiluted collected conditioned media before the 1:1 dilution used for C28/I2 treatment. (**D**) RT-qPCR analysis of ACAN, COL2A1, IL6, MMP13, PTGS2, and ADAMTS5 in C28/I2 cells treated with Control-CM, matched cytokines, or Primed-CM. The matched-cytokine condition consisted of the Control-CM treatment mixture supplemented with recombinant TNF-α and IL-6 so that the final total concentrations matched those calculated for the 1:1 diluted Primed-CM. (**E**) Representative western blots and β-actin-normalized quantification of ADAMTS-5, COX-2, and MMP-13 under the same three treatment conditions. Data are presented as mean ± SD from five independent biological experiments, and each point represents one independent experiment. Two-condition comparisons in panels (**A**–**C**) used two-sided paired Student’s *t*-tests. Three-condition comparisons in panels (**D**,**E**) used one-way repeated-measures ANOVA followed by Šídák-adjusted planned comparisons. RT-qPCR tests were performed on paired ΔCt values, whereas 2^−ΔΔCt^ values are plotted. Exact *p* values are shown above the brackets. Abbreviations: ANOVA, analysis of variance; ns, not significant; ADAMTS-5, a disintegrin and metalloproteinase with thrombospondin motifs 5; CM, conditioned medium; COX-2, cyclooxygenase-2; Ct, cycle threshold; ELISA, enzyme-linked immunosorbent assay; IκBα, inhibitor of nuclear factor-κB alpha; IL-6, interleukin-6; MMP-13, matrix metalloproteinase-13; NF-κB, nuclear factor-κB; Control-CM, control conditioned medium; Primed-CM, primed conditioned medium; PBS, phosphate-buffered saline; RT-qPCR, reverse transcription quantitative polymerase chain reaction; SD, standard deviation; STAT3, signal transducer and activator of transcription 3; TNF-α, tumor necrosis factor-α.

To examine the functional plausibility of this leukocyte-shared, myeloid-enriched inflammatory framework, we used an in vitro THP-1/C28/I2 conditioned-medium system as a proof-of-concept model of immune–chondrocyte crosstalk. THP-1 cells were primed with tumor necrosis factor-α (TNF-α) plus interleukin-6 (IL-6), and paired Control and Primed conditions were generated within each of five independent biological experiments. Priming increased inflammatory and chemokine-related transcripts, increased the p-STAT3/total STAT3 and p-p65/total p65 ratios, and reduced IκBα abundance, whereas total STAT3 and total p65 abundance remained unchanged (Figure 6A,B).

Because THP-1 priming was performed with exogenous TNF-α and IL-6, we quantified both cytokines in the final conditioned media after priming, phosphate-buffered saline (PBS) washing, medium replacement, and 24 h of conditioning. TNF-α concentrations increased from 14.6 ± 2.3 pg/mL in control conditioned medium (Control-CM) to 149.7 ± 13.9 pg/mL in primed conditioned medium (Primed-CM), whereas IL-6 concentrations increased from 22.6 ± 4.5 pg/mL to 217.8 ± 16.9 pg/mL, respectively (both *p* < 0.0001; *n* = 5 independent experiments; Figure 6C). These concentrations remained substantially lower than the initial 10 ng/mL priming concentrations.

To determine the extent to which TNF-α and IL-6 at the measured final concentrations could reproduce the C28/I2 response, we generated a background-matched cytokine add-back condition. The Control-CM treatment mixture was supplemented with recombinant TNF-α and IL-6 so that the final total concentrations matched those calculated for the 1:1 diluted Primed-CM. Compared with Control-CM, the matched-cytokine condition partially suppressed ACAN and COL2A1 and increased MMP13, COX2, and ADAMTS5 expression, whereas the increase in IL6 did not reach statistical significance. Primed-CM induced further suppression of ACAN and COL2A1 and greater induction of IL6, MMP13, COX2, and ADAMTS5 than the matched-cytokine condition (Figure 6D).

Western blotting showed a corresponding graded response. The matched-cytokine condition increased ADAMTS-5, COX-2, and MMP-13 abundance relative to Control-CM, whereas Primed-CM produced significantly greater induction of all three proteins than the matched-cytokine condition (Figure 6E).

These findings indicate that TNF-α and IL-6 present in the final conditioned medium contribute to the C28/I2 inflammatory and matrix-catabolic response but do not fully reproduce the stronger response induced by Primed-CM. The additional effect is therefore interpreted as a property of the composite primed myeloid-conditioned milieu under the tested in vitro conditions; the present experiment does not identify the specific additional mediators responsible for this difference.

## 3. Discussion

This study identifies molecular convergence between frailty-associated skeletal muscle and OA joint tissues through a leukocyte-shared and myeloid-enriched inflammatory program. This shared bulk transcriptomic program provides a molecular entry point into the clinical overlap between frailty and OA. Although GSE117525 profiles vastus lateralis skeletal muscle rather than circulating leukocytes, the enrichment of leukocyte migration and cytokine response pathways indicates an immune-associated transcriptional pattern that converges with OA synovial pathology. Consistent with this interpretation, the tissue-deconvolution sensitivity analysis indicated that variation in immune-cell abundance contributed to, but did not fully account for, the bulk shared-signature signal, with greater attenuation in frail skeletal muscle than in OA synovium. The preservation of effect direction for all 16 shared genes across adjusted GSE117525 models supports the robustness of the frailty-associated component to the measured demographic and clinical covariates. Biologically, frail skeletal muscle can generate a sterile inflammatory niche characterized by damage-associated molecular patterns (DAMPs) and myokine signaling [37,38]. Because circulating monocytes continuously survey the vascular compartment, we propose that sustained exposure to a frailty-associated inflammatory milieu may promote the inflammatory priming of these cells. Following recruitment into synovial or meniscal tissues, such primed myeloid cells could plausibly contribute to the migratory and inflammatory components represented by Hub-15. This interpretation remains hypothesis-generating and should not be considered evidence of formal trained immunity.

The PPI-derived Hub-15 refines this overlap into a focused inflammatory module integrating leukocyte identity, myeloid function, immediate-early responses, and cytokine signaling. F13A1 was the only shared gene displaying divergent directional effects between the frailty-associated muscle and OA synovial datasets. F13A1 encodes factor XIII-A, a transglutaminase implicated in alternatively activated macrophage states [39,40]. Its divergent behavior may reflect context-specific differences between impaired tissue repair in frail muscle and active, albeit pathological, matrix remodeling in OA synovium.

At the single-cell level, Hub-15 appears to mark a leukocyte-shared inflammatory response, with its highest absolute scores observed in monocyte/macrophage-like cells. Monocytes and macrophages, therefore, represent plausible contributors to the local expression of this shared inflammatory program. Extending this framework to tissue remodeling, our donor-corrected meniscal analysis indicated that the dominant OA-associated signal was a redistribution among meniscal structural states—specifically, a marked reduction in inflammatory/stress cells and an expansion of fibrochondrocyte-like cells—rather than uniform within-state expression changes alone. These findings indicate that structural-state redistribution is a prominent OA-associated feature within the meniscal compartment.

Exploratory CellChat analysis nominated candidate myeloid-to-structural communication axes, although none remained significant after multiple-testing correction. These findings were complemented by the THP-1/C28/I2 conditioned-medium model, which provided a separate system-level proof-of-concept. The background-matched cytokine add-back control indicated that TNF-α and IL-6 at the final concentrations present in the 1:1 diluted Primed-CM contributed to the C28/I2 response but did not fully reproduce the stronger inflammatory and matrix-catabolic changes induced by Primed-CM. Because the add-back condition shared the same Control-CM background, the remaining difference is consistent with additional components or signaling interactions within the primed conditioned milieu, although the present experiment does not identify the specific mediators responsible. These findings support the biological competence of a composite primed myeloid-conditioned inflammatory milieu to induce OA-relevant responses in C28/I2 cells under the tested in vitro conditions, although this approach does not validate any individual CellChat-nominated signaling axis. A recent study reported that acetyl-CoA–FOXM1 signaling promotes chondrocyte senescence and OA progression [7]. Although not examined here, TNF-α/IL-6-associated inflammatory conditioning may interact with this intrinsic metabolic program; acetyl-CoA levels, FOXM1 expression, and canonical senescence markers were not measured in the present study.

Several limitations warrant emphasis. First, the analyses rely on unmatched, retrospective public cohorts differing in tissue source, platform, and metadata depth. Consequently, although the findings identify molecular convergence, this cross-sectional design cannot establish a direct causal pathway from frailty to OA or determine the temporal directionality of any systemic-to-local relationship. Second, the single-cell localization and donor-level structural-state analyses included relatively small numbers of biological donors, including five frail and six healthy older donors in GSE157007 and seven normal and six OA donors in the meniscal dataset. The modest donor-level sample size reduced statistical power and effect-size precision, underscoring the need for larger, deeply phenotyped cohorts. Third, our functional validation lacks primary-cell and in vivo validation. Although the composite conditioned-medium approach provided an in vitro proof-of-concept, immortalized THP-1 and C28/I2 cell lines cannot fully recapitulate primary monocytes obtained from individuals with frailty or patient-derived OA chondrocytes and meniscal fibrochondrocytes. Future mechanistic validation should include primary monocytes or macrophages obtained from individuals with frailty, patient-derived OA chondrocytes and meniscal fibrochondrocytes, macrophage–structural-cell co-cultures, and appropriate in vivo OA models, including the destabilization of the medial meniscus (DMM) model. Finally, the CellChat inferences remain computational and will require targeted molecular perturbation for definitive validation.

Future studies should test the Hub-15 program in paired skeletal muscle, blood, and joint tissues from the same individuals in larger, clinically characterized frailty and OA cohorts. Single-cell transcriptomics or cellular indexing of transcriptomes and epitopes by sequencing (CITE-seq), together with targeted flow cytometry, spatial transcriptomics, and multiplex imaging, will be important for determining whether classical CD14^+^CD16^−^, intermediate CD14^+^CD16^+^, or non-classical CD14^lowCD16+^ circulating monocytes preferentially express the Hub-15-associated inflammatory program.

In summary, our multimodal analysis identifies a leukocyte-shared, myeloid-enriched inflammatory program that converges across independent frailty and OA datasets. This study provides a hypothesis-generating framework for investigating immune-structural crosstalk in age-related musculoskeletal vulnerability, while acknowledging that a direct causal link between frailty and OA remains to be formally established.

## 4. Materials and Methods

### 4.1. Study Design and Data Sources

This study integrated public bulk transcriptomic datasets, public single-cell RNA sequencing datasets and in vitro conditioned-medium experiments. Frailty-related bulk expression data were obtained from GSE117525, a human vastus lateralis skeletal muscle dataset [41]; baseline untrained older samples included healthy older controls (*n* = 32) and frail older adults (*n* = 30). OA-related bulk expression data were obtained from GSE55235 [42], including healthy synovium (*n* = 10) and OA synovium (*n* = 10), with rheumatoid arthritis samples excluded. Peripheral immune single-cell data were obtained from GSE157007 [30]; the updated quality-control object used for donor-aware analysis contained 91,538 cells from 14 donors, of which 90,185 cells had one of the ten displayed major-class annotations used in Figure 3A–C. OA knee-tissue single-cell data were obtained from GSE220243 [31]. The meniscal structural-cell endpoint analysis included 71,196 cells from 14 regional samples representing 13 donors (8 normal samples from 7 donors and 6 OA samples from 6 donors), and the myeloid-to-structural communication compartment included 73,271 cells from the same 14 samples and 13 donors. GSM6797160 and GSM6797161 were vascular and avascular samples from the same Normal Donor1. Public datasets were de-identified resources from the Gene Expression Omnibus (GEO), and the experimental component used commercially available cell lines; no new human participants or newly collected human tissue were included. Dataset-level inclusion criteria and analysis roles are summarized in Table S1 and Supplementary Note S1.

### 4.2. Bulk Transcriptomic Data Acquisition and Preprocessing

Processed GEO series-matrix expression files were imported into R. For GSE117525, GPL20880 annotation was used, and gene-level expression was summarized for 19,525 features. For GSE55235, GPL96/Affymetrix Human Genome U133A probes were mapped to official gene symbols using hgu133a.db, and 13,039 gene-level features were retained after probe-to-gene aggregation. When multiple probes mapped to the same gene, expression values were aggregated at the gene level. GSE55235 differential analysis used the log2-transformed expression matrix and the log2-scale DEG file DEG_limma_GSE55235_FC1.5_log2.csv; the unlogged raw-scale DEG output was not used for interpretation.

### 4.3. Differential Expression Analysis

Differential expression analysis was performed separately for frail versus healthy older vastus lateralis samples and OA versus healthy control synovium using limma linear modeling with empirical Bayes moderation [43]. For GSE117525, the primary model used a no-intercept group-only design (~0 + group_list) and the contrast frail versus healthy older (treat—control), with Benjamini–Hochberg FDR < 0.05 and |log2FC| > log2(1.5). Because aging- and frailty-associated skeletal muscle transcriptomic shifts are often subtle, and the primary contrast yielded a limited number of DEGs, a 1.5-fold threshold was selected to retain biologically interpretable inflammatory signals while maintaining FDR control. This yielded 65 primary DEGs (36 upregulated and 29 downregulated). Sensitivity analyses included an age/sex-adjusted model and a fuller model adjusted for age, sex, body mass index and supplement status. The age/sex-adjusted analysis yielded 69 DEGs and retained 58 of 65 primary DEGs; the fuller model yielded 45 DEGs and retained 39 of 65 primary DEGs. Among the 16 shared frailty-OA genes, 14 of 16 remained significant after age/sex adjustment, 9 of 16 remained significant after full adjustment and all 16 retained the same effect direction in both adjusted models. For GSE55235, the same group-only limma framework was used with the contrast OA versus healthy control, yielding 1510 DEGs (911 upregulated and 599 downregulated) at the same threshold. Model-specific outputs and sensitivity analyses are detailed in Table S2 and Supplementary Note S2.

### 4.4. Shared DEG Identification and Concordance Analysis

Genes significantly differentially expressed in both bulk datasets were intersected to identify shared frailty-OA DEGs. Statistical enrichment of the overlap was tested using a one-sided Fisher exact/hypergeometric test against the harmonized common measured-gene universe defined after gene-symbol annotation and dataset intersection. The final overlap contained 16 shared genes, 49 frailty-only genes and 1494 OA-only genes and yielded an odds ratio of 2.41 with *p* = 0.0036; the complete background cell of the 2 × 2 table and the gene-universe file are provided in Source Data. Concordance was visualized by plotting log2 fold changes from the frailty-associated vastus lateralis contrast against those from the OA synovial contrast, and a two-column heatmap summarized shared-gene effect sizes. The final 16 shared genes were *SELL*, *NR4A3*, *NR4A2*, *HES1*, *LYZ*, *PTPRC*, *KLF4*, *LAPTM5*, *CCL5*, *SORL1*, *APOLD1*, *F13A1*, *MNDA*, *NR4A1*, *JUNB* and *CXCL2*. To determine the extent to which the bulk shared-gene signal might reflect variations in immune-cell abundance, a tissue-deconvolution sensitivity analysis was performed independently in GSE117525 and GSE55235. MCP-counter was employed as the primary estimation method, with xCell serving as an orthogonal validation. A composite immune score was derived from the first principal component (PC1) of the standardized MCP-counter immune-lineage scores. The directional shared-signature models were subsequently re-evaluated, adjusting for this immune covariate. Notably, these deconvolution outputs were interpreted as relative enrichment scores rather than absolute cell fractions.

### 4.5. Functional Enrichment Analysis

Gene Ontology (GO) and Kyoto Encyclopedia of Genes and Genomes (KEGG) enrichment analyses were performed using clusterProfiler [44], with gene symbols mapped to Entrez Gene identifiers using org.Hs.eg.db and the Benjamini–Hochberg adjustment. Three scopes were retained. First, strict GO enrichment of the 16 shared DEGs used the common measured-gene universe. Second, strict GO enrichment was performed for the final PPI-derived Hub-15 module. Third, GO and KEGG enrichment of the STRING-expanded interaction neighborhood was used for the main network-level pathway displays because term counts exceeded the strict input-set sizes. The *Homo sapiens* STRING network was constructed at a combined score >= 0.4 [45], yielding 115 nodes after interaction-neighborhood expansion. Centrality was calculated with igraph using degree, betweenness and harmonic/closeness measures [46]. The final 15 hubs were prioritized by degree, with cross-metric rank consistency used as a robustness check. Thirteen shared genes were retained and IL6, and FOS were added as STRING-derived hubs; APOLD1 was absent from the expanded network, and HES1 and SORL1 fell below the final top-15 cutoff. The strict shared-16, strict Hub-15 and STRING-expanded scopes are cross-referenced in Table S4A–H and Supplementary Notes S3 and S4.

### 4.6. Single-Cell RNA Sequencing Data Processing

Raw count matrices from GSE157007 [30] and GSE220243 [31] were imported into Seurat [32]. For GSE157007, cells with 200–5000 detected RNA features and a mitochondrial transcript proportion < 15% were retained. Data were normalized with LogNormalize using a scale factor of 10,000, the top 2000 highly variable genes were selected by the variance-stabilizing transformation (vst) method, and donor/sample integration was performed with Harmony [33]. Principal component analysis (PCA) and uniform manifold approximation and projection (UMAP) used 30 dimensions, graph clustering used dimensions 1–30 at a resolution of 0.5, and stochastic steps used a fixed local seed of 42 without altering the global random state. For GSE220243, the resolved OA endpoint object was used. Meniscal structural analyses were restricted to cells with valid disease status, corrected structural-state annotation and meniscal tissue identity where tissue metadata were available; the communication analysis additionally retained the defined myeloid sender compartment.

### 4.7. Cell-Type and Cell-State Annotation

Clusters were initially annotated using SingleR with BlueprintEncodeData and HumanPrimaryCellAtlasData references [34], followed by manual refinement using canonical markers. The ten displayed GSE157007 immune/hematopoietic classes were CD4 T cells, CD8 T cells, natural killer (NK) cells, unconventional T/NK cells, B cells, plasma cells, monocytes/macrophage-like cells, dendritic cells, megakaryocyte/platelet cells and erythroid cells. Pseudobulk coverage was evaluated across 12 configured classes by additionally retaining cycling and hematopoietic stem and progenitor cell (HSPC)-like categories where present. GSE220243 meniscal structural states were homeostatic, PRG4+ superficial, inflammatory/stress, catabolic, fibrochondrocyte-like and stromal fibroblast. A representative 32-gene marker panel was used in the supplementary structural-state dot plot, with gene-wise scaled mean expression encoded by color and the percentage of expressing cells encoded by point area.

### 4.8. Shared Inflammatory Module Scoring

The primary downstream module was the PPI-derived 15-gene hub inflammatory/migration module generated from the shared frailty-OA PPI network [45,46]. It comprised *PTPRC*, *CCL5*, *SELL*, *CXCL2*, *MNDA*, *NR4A1*, *NR4A2*, *JUNB*, *LAPTM5*, *LYZ*, *KLF4*, *NR4A3*, *F13A1*, *IL6* and *FOS*. Thirteen genes overlapped the shared-16 set, whereas *IL6* and *FOS* were STRING-expanded hubs; *HES1*, *SORL1* and *APOLD1* were not included in the final Hub-15. Module scores were calculated on the RNA assay using Seurat AddModuleScore [32] with detected module genes and a fixed local seed of 42. For visualization, Hub-15-high cells were defined as the top 5% of scores among the 90,185 major-class-annotated GSE157007 cells (*n* = 4510) or within the complete meniscal structural atlas. Donor-aware GSE157007 inference used raw-count pseudobulks rather than AddModuleScore values, whereas GSE220243 state-specific summaries used donor-level median Hub-15 scores. As a sensitivity analysis, the principal module-dependent analyses in GSE157007 and GSE220243 were repeated using the complete original shared-16 signature. This evaluation retained the exact quality-controlled objects, cell annotations, donor definitions, coverage thresholds, module-scoring procedures, and donor-level statistical frameworks used for the primary Hub-15 analyses. Module definitions and primary/sensitivity-analysis results are summarized in Supplementary Note S5 and Supplementary Tables S4H and S10.

### 4.9. Donor-Aware GSE157007 Module Analysis

For GSE157007 inference, the donor was the biological replicate and Frail versus Healthy older was the primary contrast; Young donors were retained only for atlas and age-context visualization. Raw unique molecular identifier (UMI) counts were summed within donor × major immune-cell class. Donor-class units required at least 20 cells, and a class required at least three donors in each comparison group. Six of the 12 configured classes were evaluable. Gene-level differential expression used edgeR quasi-likelihood negative-binomial models with trimmed mean of M-values (TMM) normalization and robust dispersion estimation. The Hub-15 module was tested with the limma-voom fry self-contained gene-set test, and module-level *p* values were BH-adjusted across evaluable cell classes. As a descriptive effect-size companion, gene-wise standardized pseudobulk log2 counts-per-million (logCPM) values were averaged across detected Hub-15 genes for each donor-class pseudobulk; group mean differences were summarized with 2000-replicate bootstrap 95% confidence intervals. Classes failing cell or donor thresholds were reported as insufficient coverage rather than assigned a zero effect. Donor-median AddModuleScore summaries were retained for descriptive myeloid age-context and state-ordering panels, not as the primary Frail versus Healthy older class-specific test.

### 4.10. Myeloid State Ordering Analysis

Myeloid-lineage cells were extracted from GSE157007 and analyzed using the precomputed marker-defined myeloid state-ordering score stored in the resolved analysis workflow. Cells and donor-binned median Hub-15 values were projected along this score, and black summaries show mean ± standard error of the mean (SEM) across donor bins. No artificial random ordering was generated when a valid score was unavailable. The axis was interpreted as transcriptional-state organization rather than temporal progression, lineage tracing or cell trafficking.

### 4.11. Meniscal Structural State Analysis

For GSE220243, meniscal structural cells were retained when they had valid corrected structural-state annotations, disease status and meniscal tissue identity where tissue metadata were available. The atlas included 71,196 cells from 14 regional samples representing 13 independent donors: 8 Normal regional samples from 7 donors and 6 OA samples from 6 donors. GSM6797160 and GSM6797161 represented avascular and vascular regions from the same Normal Donor1. All 14 samples were retained for atlas visualization and sample-structure quality control. For inferential analyses, the two Normal Donor1 regions were prespecified for pooling before donor-level aggregation, resulting in 7 Normal and 6 OA donors. Structural states included homeostatic, PRG4+ superficial, inflammatory/stress, catabolic, fibrochondrocyte-like and stromal fibroblast. Hub-15-high cells were defined as the global top 5% of structural-atlas module scores for visualization; 90%, 95% and 97.5% threshold-sensitivity outputs were retained in Supplementary Source Data.

### 4.12. OA-Associated State-Shift Analysis

OA-associated shifts in structural-state proportions and continuous state-specific Hub-15 scores were tested using independent donors. Regional samples were combined within donors before calculating state fractions or donor-state score summaries; specifically, GSM6797160 and GSM6797161 were pooled as Normal Donor1. Final comparisons used 7 Normal and 6 OA donors. Structural-state fraction effects were displayed as OA-Normal differences in group mean fractions with 2000-replicate donor-level bootstrap 95% confidence intervals; Wilcoxon rank-sum *p* values were BH-adjusted across the six states. State-specific Hub-15 inference used the donor-level median score within each state, with 2000-replicate bootstrap confidence intervals, Wilcoxon tests and BH correction across states. The resolved object contained a precomputed structural state-ordering score; no random fallback score was permitted. Ordering and Low/Mid/High segment summaries were interpreted descriptively as transcriptional organization rather than temporal lineage tracing.

### 4.13. Exploratory Cell-Cell Communication Inference

Figure 5 presents inferred myeloid-to-meniscal structural communication potential. CellChat v1.6.1 and the human CellChatDB were applied separately to Normal and OA groups [36], with myeloid cells as senders and homeostatic, PRG4+ superficial, inflammatory/stress, catabolic, fibrochondrocyte-like and stromal fibroblast cells as receivers. Secreted signaling, extracellular matrix–receptor and cell–cell contact interactions were retained. The full communication compartment included 43,247 Normal cells from 8 regional samples representing 7 donors and 30,024 OA cells from 6 samples representing 6 donors. Absolute Normal and OA network plots used identical probability scales; OA-Normal differences were summarized at receiver, pathway and ligand–receptor pair levels as group-level communication-potential outputs. Donor-level expression-proxy support used the validated CellChat-oriented 29,449-cell Seurat object, comprising 2075 myeloid sender cells and six structural receiver states. GSM6797160 and GSM6797161 were pooled as Normal Donor1 before donor summaries were calculated. For each donor and selected axis, normalized log1p expression was transformed to a linear scale; mean ligand expression in myeloid sender cells was multiplied by mean receptor expression in the target structural state. For multisubunit receptors, the receptor term was the geometric mean of subunit-specific means. A donor-axis value required at least 20 sender cells and 20 receiver-state cells. These proxies provide expression-level plausibility support and do not demonstrate secretion, binding, receptor activation or causal signaling. Further details regarding the CellChat inference boundaries, donor-level expression-proxy construction, donor pooling, and coverage criteria are provided in Supplementary Note S6.

### 4.14. Cell Culture and THP-1 Inflammatory Priming

The human monocytic cell line THP-1 (American Type Culture Collection, TIB-202) was maintained in RPMI-1640 medium (Gibco) supplemented with 10% fetal bovine serum (FBS) and 1% penicillin-streptomycin at 37 °C in a humidified incubator containing 5% CO_2_. The human chondrocyte cell line C28/I2 (Merck Millipore, Burlington, MA, USA; catalog no. SCC043) was cultured in high-glucose Dulbecco’s modified Eagle medium (DMEM) supplemented with 10% FBS and 1% penicillin-streptomycin under the same conditions. Cells were used at 70–80% confluence, and both cell lines were routinely tested and confirmed to be free of mycoplasma contamination before experiments. To model low-intensity inflammatory stimulation relevant to a frailty-like inflammatory milieu, THP-1 cells were treated for 24 h with recombinant human TNF-α (10 ng/mL; PeproTech (Cranbury, NJ, USA), 300-01A) plus recombinant human IL-6 (10 ng/mL; PeproTech, 200-06). Vehicle-treated cells served as paired controls. After stimulation, cells were washed twice with PBS, transferred to fresh complete medium and allowed to rest for 2 h before downstream analyses.

### 4.15. THP-1 Conditioned Medium and C28/I2 Treatment

After priming and washing, THP-1 cells were cultured for an additional 24 h in fresh complete medium. Supernatants were collected, cleared by centrifugation to remove cell debris, and filtered through a 0.22-μm membrane. Conditioned medium from vehicle-treated THP-1 cells was designated Control-CM, whereas conditioned medium from TNF-α/IL-6-primed THP-1 cells was designated Primed-CM. For recipient-cell experiments, Control-CM and Primed-CM were each mixed 1:1 with fresh C28/I2 culture medium. A third background-matched cytokine add-back condition was generated using the Control-CM treatment mixture as described below. C28/I2 cells were exposed for 24 h before reverse transcription quantitative polymerase chain reaction (RT-qPCR) analysis and for 48 h before western blotting.

### 4.16. ELISA Quantification and Background-Matched Cytokine Add-Back Control

To quantify TNF-α and IL-6 present in the final conditioned media, Control-CM and Primed-CM were analyzed using human-specific Quantikine enzyme-linked immunosorbent assay (ELISA) kits according to the manufacturers’ instructions. Human TNF-α was measured using the Human TNF-α Quantikine ELISA Kit (R&D Systems/Bio-Techne, Minneapolis, MN, USA; catalog no. DTA00D), and human IL-6 was measured using the Human IL-6 Quantikine ELISA Kit (R&D Systems/Bio-Techne; catalog no. D6050). Absorbance was measured at 450 nm, with wavelength correction at 540 or 570 nm where available, and cytokine concentrations were interpolated from standard curves. The mean minimum detectable dose was 4.00 pg/mL for the TNF-α assay and <0.70 pg/mL for the IL-6 assay. All measured samples were above the corresponding assay detection limits.

To distinguish the effects of TNF-α and IL-6 from those of the broader conditioned milieu, a background-matched cytokine add-back control was generated. Control-CM was mixed 1:1 with fresh C28/I2 culture medium, matching the medium composition used for the Control-CM and Primed-CM treatment groups. Recombinant human TNF-α (PeproTech, Thermo Fisher Scientific, Cranbury, NJ, USA; catalog no. 300-01A) and recombinant human IL-6 (PeproTech, Thermo Fisher Scientific; catalog no. 200-06) were then added to this Control-CM treatment mixture.

The target final total cytokine concentrations were calculated from the ELISA measurements of Primed-CM after accounting for the 1:1 dilution used for C28/I2 treatment and were approximately 74.9 pg/mL for TNF-α and 108.9 pg/mL for IL-6. Because the 1:1 diluted Control-CM already contained approximately 7.3 pg/mL TNF-α and 11.3 pg/mL IL-6, recombinant TNF-α and IL-6 were added at final concentrations of approximately 67.6 pg/mL and 97.6 pg/mL, respectively. The resulting condition was designated the matched-cytokine condition.

The three recipient-cell treatment groups were therefore: (i) Control-CM, consisting of Control-CM mixed 1:1 with fresh C28/I2 medium; (ii) matched cytokines, consisting of the Control-CM treatment mixture supplemented with recombinant TNF-α and IL-6 to match the final total concentrations calculated for diluted Primed-CM; and (iii) Primed-CM, consisting of Primed-CM mixed 1:1 with fresh C28/I2 medium. All three conditions were prepared and applied in parallel within each independent biological experiment. C28/I2 cells were exposed for 24 h before RT-qPCR analysis and for 48 h before western blotting.

### 4.17. RT-qPCR

Total RNA was extracted using TRIzol Reagent (Invitrogen, Thermo Fisher Scientific, Waltham, MA, USA; catalog no. 15596026) according to the manufacturer’s instructions. Genomic DNA (gDNA) was removed, and complementary DNA (cDNA) was synthesized using the PrimeScript RT reagent Kit with gDNA Eraser (Takara Bio, Shiga, Japan; catalog no. RR047A). The genomic-DNA removal reaction was performed at 42 °C for 2 min, followed by reverse transcription at 37 °C for 15 min and enzyme inactivation at 85 °C for 5 s. Quantitative real-time PCR was performed using TB Green Premix Ex Taq II (Tli RNaseH Plus) (Takara Bio; catalog no. RR820A) on an Applied Biosystems QuantStudio 5 Real-Time PCR System (Thermo Fisher Scientific). The amplification program comprised initial denaturation at 95 °C for 30 s, followed by 40 cycles of 95 °C for 5 s and 60 °C for 30 s, after which melting-curve analysis was performed to verify amplification specificity. THP-1 targets were IL1B, TNF, NFKBIA, SOCS3, CXCL8, CCL5 and CXCL2. C28/I2 targets were MMP13, ADAMTS5, IL6, PTGS2, COL2A1, ACAN. ACTB was used as the internal reference gene. Relative expression was calculated using the 2^−ΔΔCt^ method, with the paired control condition used as the calibrator. Statistical comparisons were performed on paired ΔCt values, whereas 2^−ΔΔCt^ values were used for graphical presentation. All human forward and reverse primer sequences are provided in Table S9.

### 4.18. Western Blotting

The cells were lysed in radioimmunoprecipitation assay (RIPA) buffer (Beyotime, Shanghai, China; catalog no. P0013C) supplemented with 1 mM phenylmethylsulfonyl fluoride. Protein concentrations were determined using a bicinchoninic acid (BCA) Protein Assay Kit (Beyotime; catalog no. P0012). Equal amounts of protein (25 μg per lane) were separated by 10–12% sodium dodecyl sulfate–polyacrylamide gel electrophoresis (SDS-PAGE) and transferred onto polyvinylidene difluoride (PVDF) membranes. Membranes were blocked with 5% bovine serum albumin (BSA) for 1 h at room temperature, incubated with primary antibodies overnight at 4 °C, washed with Tris-buffered saline containing Tween 20 (TBST) and incubated with the appropriate horseradish peroxidase (HRP)-conjugated secondary antibodies for 1 h at room temperature. Primary antibodies included phospho-STAT3 (Tyr705; Cell Signaling Technology (Danvers, MA, USA), #9145; 1:1000), total STAT3 (Cell Signaling Technology, #9139; 1:1000), phospho-NF-κB p65 (Ser536; Cell Signaling Technology, #3033; 1:1000), total NF-κB p65 (Cell Signaling Technology, #8242; 1:1000), IκBα (Cell Signaling Technology, #4812; 1:1000), MMP-13 (Abcam (Cambridge, UK), ab39012; 1:1000), ADAMTS-5 (Abcam, ab182795; 1:1000), COX-2 (Abcam, ab179800; 1:2000) and β-actin (Cell Signaling Technology, #4970; 1:5000). Signals were visualized using enhanced chemiluminescence and quantified in ImageJ (version 1.54). For densitometric analysis, phosphorylated STAT3 was normalized to total STAT3, and phosphorylated p65 was normalized to total p65. Total STAT3, total p65, IκBα, ADAMTS-5, COX-2, and MMP-13 were normalized to β-actin. For each independent biological experiment, normalized values were expressed relative to the corresponding paired control condition. For C28/I2 analyses, ADAMTS-5, COX-2, and MMP-13 were normalized to β-actin. Densitometric comparisons were performed among the paired Control-CM, matched-cytokine, and Primed-CM conditions generated within each independent biological experiment.

### 4.19. Statistical Analysis

Statistical analyses were performed in R. Bulk differential-expression analyses used limma empirical Bayes models with Benjamini–Hochberg correction, and DEGs were defined as FDR < 0.05 and |log2FC| > log2(1.5). The shared-DEG overlap was evaluated by one-sided Fisher exact/hypergeometric testing against the harmonized common measured-gene universe. Enrichment analyses used BH-adjusted *p* < 0.05. For GSE157007, raw UMI counts were aggregated within donor × major-cell-class pseudobulks; gene-level effects used edgeR quasi-likelihood models, and the Hub-15 gene set used limma-voom fry, with BH correction across evaluable classes. Descriptive standardized pseudobulk-score differences were accompanied by 2000-replicate bootstrap 95% confidence intervals. GSE220243 structural-state analyses used 7 Normal and 6 OA donors after pooling GSM6797160 and GSM6797161 as Normal Donor1; state-fraction and donor-median Hub-15 comparisons used Wilcoxon rank-sum tests, 2000-replicate bootstrap confidence intervals and BH correction across six states. Figure 5 CellChat receiver, pathway and ligand–receptor contrasts were group-level inferred communication-potential summaries. Donor-level expression-proxy comparisons used independent donors, two-sided Wilcoxon rank-sum tests, 2000-replicate bootstrap confidence intervals for OA-Normal mean differences and BH correction across the six prioritized axes.

All in vitro experiments were performed across five independent biological experiments. Within each experiment, paired treatment conditions were derived from the same cell preparation and processed in parallel. Data are presented as mean ± standard deviation (SD), and each plotted point represents one independent biological experiment rather than a technical replicate. Two-condition comparisons in Figure 6A–C were performed using two-sided paired Student’s *t*-tests. For the three-condition C28/I2 experiments in Figure 6D,E, paired measurements were analyzed using one-way repeated-measures analysis of variance, followed by Šídák-adjusted planned pairwise comparisons. RT-qPCR analyses were performed on ΔCt values, whereas 2^−ΔΔCt^ values are plotted. Western blot analyses were performed on normalized densitometric values. Phosphorylated STAT3 and phosphorylated p65 were analyzed as p-STAT3/total STAT3 and p-p65/total p65 ratios, respectively; total STAT3, total p65, IκBα, ADAMTS-5, COX-2, and MMP-13 were normalized to β-actin. Exact *p* values are shown in Figure 6, with Šídák-adjusted *p* values reported for the three-condition comparisons.

### 4.20. Reproducibility and Software

Analyses were performed throughout the project using R environments documented by sessionInfo files. The Figure 2 enrichment workflow used R 4.5.1 with clusterProfiler 4.16.0, org.Hs.eg.db 3.21.0, AnnotationDbi 1.70.0, enrichplot 1.28.4, ggplot2 4.0.1, dplyr 1.1.4 and readr 2.1.5. The GSE157007 pseudobulk workflow used R 4.5.1 with Seurat 5.3.0, SeuratObject 5.2.0, edgeR 4.6.3, limma 3.64.3, Matrix 1.7-4, ggplot2 4.0.1 and dplyr 1.1.4. The Figure 4 and Figure 5 GSE220243 workflows used R 4.3.3; key packages included Seurat 5.3.0, SeuratObject 5.2.0, slingshot 2.10.0, harmony 2.0.2, CellChat 1.6.1, ggplot2 4.0.3, dplyr 1.2.1, patchwork 1.3.2 and future 1.70.0. Random seeds were fixed or locally isolated at 42 in single-cell, bootstrap and figure-generation workflows where stochastic steps were used. Source-data tables include figure-specific file indices linking each panel to the corresponding source-data file. Complete workflow-specific software and package versions are provided in Table S8.

### 4.21. Code Availability

Custom R scripts and workflow-specific session information are available from the corresponding author upon reasonable request. Requests should be directed to shizhibin@xjtu.edu.cn (Z.S.).

### 4.22. Ethics Statement

This study used publicly available, de-identified human transcriptomic datasets and commercially available cell lines. No new human participants were recruited, and no new human tissue samples were collected for this study. Therefore, additional institutional review board approval and informed consent were not required for the secondary public-data analyses or the cell-line experiments.

## Data Availability

The public datasets analyzed in this study are available from the Gene Expression Omnibus under accession numbers GSE117525, GSE55235, GSE157007 and GSE220243. Source tables generated during the bulk, enrichment, single-cell pseudobulk/module-score, structural-state and CellChat analyses for Figure 1, Figure 2, Figure 3, Figure 4 and Figure 5 are provided in the Supplementary Tables and accompanying Source Data files. The GSE220243 metadata distinguish 14 regional samples from 13 independent donors; GSM6797160 and GSM6797161 derive from the same Normal Donor1 and were pooled for the final Figure 4 and Figure 5 donor-level analyses. Human RT-qPCR primer sequences are provided in Table S9. Full-length uncropped immunoblots underlying Figure 6 are provided separately with the journal submission.

## Supplementary Materials

The following supporting information can be downloaded at https://www.mdpi.com/article/10.3390/ijms27156743/s1.

## References

[1] GBD 2021 Osteoarthritis Collaborators (2023). Global, regional, and national burden of osteoarthritis, 1990–2020 and projections to 2050: A systematic analysis for the Global Burden of Disease Study 2021. Lancet Rheumatol..

[2] Katz J.N., Arant K.R., Loeser R.F. (2021). Diagnosis and treatment of hip and knee osteoarthritis: A review. JAMA.

[3] Yao Q., Wu X., Tao C., Gong W., Chen M., Qu M., Zhong Y., He T., Chen S., Xiao G. (2023). Osteoarthritis: Pathogenic signaling pathways and therapeutic targets. Signal Transduct. Target. Ther..

[4] Hunter D.J., Bierma-Zeinstra S. (2019). Osteoarthritis. Lancet.

[5] Han Z., Wang K., Ding S., Zhang M. (2024). Cross-talk of inflammation and cellular senescence: A new insight into the occurrence and progression of osteoarthritis. Bone Res..

[6] Knights A.J., Redding S.J., Maerz T. (2023). Inflammation in osteoarthritis: The latest progress and ongoing challenges. Curr. Opin. Rheumatol..

[7] Song J., Kim E.H., Yang J.H., Kim D., Robby A.I., Kim S.-A., Park S.Y., Ryu J.H., Jin E.-J. (2023). Upregulated FOXM1 stimulates chondrocyte senescence in Acot12^−/−^Nudt7^−/−^ double knockout mice. Theranostics.

[8] Fried L.P., Tangen C.M., Walston J., Newman A.B., Hirsch C., Gottdiener J., Seeman T., Tracy R., Kop W.J., Burke G. (2001). Frailty in older adults: Evidence for a phenotype. J. Gerontol. A Biol. Sci. Med. Sci..

[9] Hoogendijk E.O., Afilalo J., Ensrud K.E., Kowal P., Onder G., Fried L.P. (2019). Frailty: Implications for clinical practice and public health. Lancet.

[10] Kim D.H., Rockwood K. (2024). Frailty in older adults. N. Engl. J. Med..

[11] Cook M.J., Verstappen S.M.M., Lunt M., O’Neill T.W. (2022). Increased frailty in individuals with osteoarthritis and rheumatoid arthritis and the influence of comorbidity: An analysis of the UK Biobank cohort. Arthritis Care Res..

[12] Cai G., Zhang Y., Wang Y., Li X., Xu S., Shuai Z., Pan F., Peng X. (2023). Frailty predicts knee pain trajectory over 9 years: Results from the Osteoarthritis Initiative. Pain Med..

[13] Huang G., Qian D., Liu Y., Qu G., Qian Y., Pei B. (2023). The association between frailty and osteoarthritis based on the NHANES and Mendelian randomization study. Arch. Med. Sci..

[14] Blanco-Reina E., Aguilar-Cano L., García-Merino M.R., Ocaña-Riola R., Valdellós J., Bellido-Estévez I., Ariza-Zafra G. (2021). Assessing prevalence and factors related to frailty in community-dwelling older adults: A multinomial logistic analysis. J. Clin. Med..

[15] Pegreffi F., Balestra A., De Lucia O., Smith L., Barbagallo M., Veronese N. (2023). Prevalence of sarcopenia in knee osteoarthritis: A systematic review and meta-analysis. J. Clin. Med..

[16] Sanchez-Lopez E., Coras R., Torres A., Lane N.E., Guma M. (2022). Synovial inflammation in osteoarthritis progression. Nat. Rev. Rheumatol..

[17] Zhu R., Fang H., Wang J., Ge L., Zhang X., Aitken D., Cai G. (2024). Inflammation as a therapeutic target for osteoarthritis: A literature review of clinical trials. Clin. Rheumatol..

[18] Griffin T.M., Scanzello C.R. (2019). Innate inflammation and synovial macrophages in osteoarthritis pathophysiology. Clin. Exp. Rheumatol..

[19] Zhang Y., Ji Q. (2023). Macrophage polarization in osteoarthritis progression: A promising therapeutic target. Front. Cell Dev. Biol..

[20] Jrad A.I.S., Trad M., Bzeih W., El Hasbani G., Uthman I. (2023). Role of pro-inflammatory interleukins in osteoarthritis: A narrative review. Connect. Tissue Res..

[21] Mushenkova N.V., Nikiforov N.G., Shakhpazyan N.K., Orekhova V.A., Sadykhov N.K., Orekhov A.N. (2022). Phenotype diversity of macrophages in osteoarthritis: Implications for development of macrophage modulating therapies. Int. J. Mol. Sci..

[22] Wang Z., Zhu P., Liao B., You H., Cai Y. (2023). Effects and action mechanisms of individual cytokines contained in PRP on osteoarthritis. J. Orthop. Surg. Res..

[23] Franceschi C., Garagnani P., Parini P., Giuliani C., Santoro A. (2018). Inflammaging: A new immune-metabolic viewpoint for age-related diseases. Nat. Rev. Endocrinol..

[24] Furman D., Campisi J., Verdin E., Carrera-Bastos P., Targ S., Franceschi C., Ferrucci L., Gilroy D.W., Fasano A., Miller G.W. (2019). Chronic inflammation in the etiology of disease across the life span. Nat. Med..

[25] Fulop T., Witkowski J.M., Olivieri F., Larbi A. (2018). The integration of inflammaging in age-related diseases. Semin. Immunol..

[26] Reitsema R.D., Kumawat A.K., Hesselink B.C., van Baarle D., van Sleen Y. (2024). Effects of ageing and frailty on circulating monocyte and dendritic cell subsets. npj Aging.

[27] De Maeyer R.P.H., Sikora J., Bracken O.V., Shih B., Lloyd A.F., Peckham H., Hollett K., Abdelhamid K., Cai W., James M. (2025). Age-associated inflammatory monocytes are increased in menopausal females and reversed by hormone replacement therapy. Aging Cell.

[28] Ji Q., Zheng Y., Zhang G., Hu Y., Fan X., Hou Y., Wen L., Li L., Xu Y., Wang Y. (2019). Single-cell RNA-seq analysis reveals the progression of human osteoarthritis. Ann. Rheum. Dis..

[29] Zhao X., Gu M., Xu X., Wen X., Yang G., Li L., Sheng P., Meng F. (2020). CCL3/CCR1 mediates CD14+CD16- circulating monocyte recruitment in knee osteoarthritis progression. Osteoarthr. Cartil..

[30] Luo O.J., Lei W., Zhu G., Ren Z., Xu Y., Xiao C., Zhang H., Cai J., Luo Z., Gao L. (2022). Multidimensional single-cell analysis of human peripheral blood reveals characteristic features of the immune system landscape in aging and frailty. Nat. Aging.

[31] Swahn H., Li K., Duffy T., Olmer M., D’Lima D.D., Mondala T.S., Natarajan P., Head S.R., Lotz M.K. (2023). Senescent cell population with ZEB1 transcription factor as its main regulator promotes osteoarthritis in cartilage and meniscus. Ann. Rheum. Dis..

[32] Stuart T., Butler A., Hoffman P., Hafemeister C., Papalexi E., Mauck W.M., Hao Y., Stoeckius M., Smibert P., Satija R. (2019). Comprehensive integration of single-cell data. Cell.

[33] Korsunsky I., Millard N., Fan J., Slowikowski K., Zhang F., Wei K., Baglaenko Y., Brenner M., Loh P.-R., Raychaudhuri S. (2019). Fast, sensitive and accurate integration of single-cell data with Harmony. Nat. Methods.

[34] Aran D., Looney A.P., Liu L., Wu E., Fong V., Hsu A., Chak S., Naikawadi R.P., Wolters P.J., Abate A.R. (2019). Reference-based analysis of lung single-cell sequencing reveals a transitional profibrotic macrophage. Nat. Immunol..

[35] Street K., Risso D., Fletcher R.B., Das D., Ngai J., Yosef N., Purdom E., Dudoit S. (2018). Slingshot: Cell lineage and pseudotime inference for single-cell transcriptomics. BMC Genom..

[36] Jin S., Guerrero-Juarez C.F., Zhang L., Chang I., Ramos R., Kuan C.H., Myung P., Plikus M.V., Nie Q. (2021). Inference and analysis of cell-cell communication using CellChat. Nat. Commun..

[37] Chazaud B. (2020). Inflammation and skeletal muscle regeneration: Leave it to the macrophages!. Trends Immunol..

[38] Wang X., Zhou L. (2022). The many roles of macrophages in skeletal muscle injury and repair. Front. Cell Dev. Biol..

[39] Törőcsik D., Bárdos H., Nagy L., Ádány R. (2005). Identification of factor XIII-A as a marker of alternative macrophage activation. Cell. Mol. Life Sci..

[40] Dull K., Fazekas F., Törőcsik D. (2021). Factor XIII-A in diseases: Role beyond blood coagulation. Int. J. Mol. Sci..

[41] Hangelbroek R.W.J., Fazelzadeh P., Tieland M., Boekschoten M.V., Hooiveld G.J.E.J., van Duynhoven J.P.M., Timmons J.A., Verdijk L.B., de Groot L.C.P.G.M., van Loon L.J.C. (2016). Expression of protocadherin gamma in skeletal muscle tissue is associated with age and muscle weakness. J. Cachexia Sarcopenia Muscle.

[42] Woetzel D., Huber R., Kupfer P., Pohlers D., Pfaff M., Driesch D., Häupl T., Koczan D., Stiehl P., Guthke R. (2014). Identification of rheumatoid arthritis and osteoarthritis patients by transcriptome-based rule set generation. Arthritis Res. Ther..

[43] Ritchie M.E., Phipson B., Wu D., Hu Y., Law C.W., Shi W., Smyth G.K. (2015). limma powers differential expression analyses for RNA-sequencing and microarray studies. Nucleic Acids Res..

[44] Wu T., Hu E., Xu S., Chen M., Guo P., Dai Z., Feng T., Zhou L., Tang W., Zhan L. (2021). clusterProfiler 4.0: A universal enrichment tool for interpreting omics data. Innovation.

[45] Szklarczyk D., Kirsch R., Koutrouli M., Nastou K., Mehryary F., Hachilif R., Gable A.L., Fang T., Doncheva N.T., Pyysalo S. (2023). The STRING database in 2023: Protein-protein association networks and functional enrichment analyses for any sequenced genome of interest. Nucleic Acids Res..

[46] Antonov M., Csardi G., Horvat S., Muller K., Nepusz T., Noom D., Salmon M., Traag V., Welles B.F., Zanini F. (2023). igraph enables fast and robust network analysis across programming languages. arXiv.

