# Supplementary material for: Cross-Tissue Transcriptomic Convergence Identifies a Leuko-Cyte-Shared, Myeloid-Enriched Inflammatory Program in Frailty and Osteoarthritis
Source: Int J Mol Sci. 2026 Jul 28;27(15):6743. doi: 10.3390/ijms27156743 (PMC13467158; doi:10.3390/ijms27156743)
Supplement: Supplementary file 1 [file ijms-27-06743-s001.zip › Supplementary files/Supplementary_Information.pdf]

Supplementary Information

Supplementary methods, notes and legends

Supplementary Note S1. Public dataset processing and sample inclusion

GSE117525 was used as a frailty-associated vastus lateralis skeletal-muscle bulk transcriptomic signature. Baseline untrained older samples were retained for the primary frail versus healthy older contrast. GSE55235 was used as the OA synovial bulk signature. GSE157007 provided peripheral immune single-cell context; the major-class-annotated atlas contained 90,185 cells from 14 donors, comprising Young (3 donors; 23,822 cells), Healthy older (6 donors; 47,444 cells) and Frail (5 donors; 18,919 cells). GSE220243 provided OA-side meniscal structural and communication context from 14 regional samples representing 13 independent donors: 8 Normal samples from 7 donors and 6 OA samples from 6 donors. GSM6797160 and GSM6797161 are avascular and vascular regions from the same Normal Donor1 and were pooled before donor-level Figure 4 inference.

Supplementary Note S2. Differential-expression and sensitivity analyses

Bulk differential expression used limma with Benjamini–Hochberg FDR control and a 1.5-fold threshold. GSE117525 sensitivity analyses included age/sex and broader covariate-adjusted models to assess robustness of the frailty-associated skeletal-muscle signal.

Supplementary Note S3. Shared16, enrichment scope and PPI-expanded analyses

The shared set contains 16 genes intersecting the frailty skeletal-muscle and OA synovial DEG signatures. Enrichment using only these 16 genes is distinct from enrichment of the STRING-expanded PPI network. Terms with gene counts greater than 16 represent network-level enrichment.

Supplementary Note S4. PPI-derived Hub-15 module

The Hub-15 module was derived from the shared-gene PPI network using centrality ranking. It overlaps 13 of the 16 shared genes and includes IL6 and FOS as STRING-expanded hub genes. HES1, SORL1 and APOLD1 are not part of the primary Hub-15 module.

Supplementary Note S5. Single-cell module scoring and state-ordering

Figures 3 and 4 use Hub-15 scores to localize the shared inflammatory/migration program at single-cell resolution. For GSE157007, formal Frail versus Healthy older inference was performed on the quality-controlled object by summing raw UMI counts within donor × major-cell-class pseudobulks. Donor-class units required at least 20 cells, and classes required at least three donors per group. Gene-level effects used edgeR quasi-likelihood models, and the Hub-15 module was tested with limma-voom fry followed by BH correction across evaluable classes. Significant coordinated upregulation was detected in CD8 T cells (BH FDR = 0.0268), NK cells (0.0244), B cells (0.0244) and monocyte/macrophage-like cells (0.0244); CD4 T and megakaryocyte/platelet cells were not significant, and the remaining classes lacked sufficient coverage. AddModuleScore outputs were retained for atlas localization and descriptive myeloid age-context. For GSE220243, structural-atlas localization used the top 5% threshold, with 90th-, 95th- and 97.5th-percentile sensitivity outputs retained. Donor-level inference pooled the two Normal Donor1 regions, yielding 7 Normal and 6 OA donors. State-ordering analyses describe transcriptional organization only and do not establish temporal lineage progression.

Supplementary Note S6. CellChat inference boundaries

Figure 5 infers myeloid-to-meniscal structural communication potential using CellChat. Receiver, pathway and ligand-receptor summaries are interpreted as computational communication hypotheses, not direct evidence of physical binding or causality. Donor-level expression-proxy support used the 29,449-cell communication object, comprising 2,075 myeloid sender cells and six structural receiver states. GSM6797160 and GSM6797161 were pooled as Normal Donor1 before donor summaries were calculated. For each donor, mean linearized ligand expression in myeloid cells was multiplied by target-state receptor expression; multisubunit receptors used the geometric mean of subunit means. Donors required at least 20 sender cells and 20 receiver cells. All six OA-Normal effects were positive, with the largest effects for FN1-ITGAV/ITGB8 and ADGRE5-CD55, but none reached BH FDR < 0.05.

The two homeostatic-receiver axes used 6 Normal versus 6 OA donors because one Normal donor did not meet the receiver-cell threshold; the remaining axes used 7 versus 6 donors.

Supplementary Note S7. Original shared-16 sensitivity analysis

The original shared-16 signature was rescored in GSE157007 and GSE220243 to assess whether the downstream single-cell findings depended on the STRING-added IL6 and FOS genes included in Hub-15. In GSE157007, the Frail-versus-Healthy older effect direction was preserved in five of the six evaluable immune-cell classes; CD8 T cells, NK cells and monocyte/macrophage-like cells remained BH-significant, whereas the B-cell effect remained similar in direction and magnitude but did not pass BH correction. In GSE220243, all six structural states retained the same OA-versus-Normal direction, the inflammatory/stress state remained the highest-scoring structural state, and Hub-15 and shared-16 scores showed strong concordance across cell-level, donor-by-state and ordering summaries. Hub-15 remains the primary downstream module, and shared-16 is reported as a robustness analysis in Table S10.

Supplementary Note S8. Bulk tissue-deconvolution sensitivity analysis

To assess whether the bulk shared-gene signal partly reflected differences in immune-cell abundance, tissue deconvolution was performed independently in GSE117525 skeletal muscle and GSE55235 synovium using MCP-counter as the primary method and xCell as an orthogonal method. A composite immune score derived from the first principal component of standardized MCP-counter immune-lineage scores was used as a sensitivity covariate. After adjustment, the directionally oriented shared-16 effect remained significant in both datasets, with 36.2% attenuation in GSE117525 and 2.3% attenuation in GSE55235. All 16 shared genes retained their within-dataset effect directions, and adjusted cross-tissue concordance remained significant. xCell-adjusted models in GSE55235 were treated as secondary because the xCell immune PC1 was highly collinear with OA status. Deconvolution scores represent relative abundance or enrichment rather than absolute cell fractions, and the analysis is interpreted as a sensitivity analysis rather than causal mediation. Principal results are summarized in Table S11.

Supplementary table legends

Table	Description
Table S1	Public datasets, sample inclusion and analysis-unit counts for Figs. 1–5.
Table S2	Bulk differential-expression results, covariate-adjusted models and threshold-sensitivity analyses.
Table S3	Shared DEG overlap, 2×2 contingency table, enrichment test and directionality.
Table S4	GO/KEGG enrichment scopes, STRING/PPI network, centrality and Hub-15 derivation.
Table S5	GSE157007 quality-controlled cell counts, major-cell-class mapping, Hub-15 detection, donor-by-cell-class pseudobulk coverage, module-level fry results, gene-level edgeR results, descriptive standardized pseudobulk effects, composition and state-ordering summaries.
Table S6	GSE220243 meniscal structural-cell counts, marker support, threshold sensitivity, donor-corrected structural-state fractions, donor-median state-specific Hub-15 effects and ordering/module-dynamics summaries. Inference used 7 Normal and 6 OA donors after pooling the two Normal Donor1 regions.
Table S7	CellChat communication-compartment QC and receiver/pathway/LR summaries, donor-level expression-proxy effect statistics, donor values, donor mapping and coverage audits. Inference uses 7 Normal and 6 OA donors after pooling the two Normal Donor1 regions.
Table S8	Software and package versions for Figs. 2–5 workflows.
Table S9	Human RT-qPCR forward and reverse primer sequences used in Fig. 6.
Table S10	Hub-15 versus original shared-16 sensitivity analyses in GSE157007 and GSE220243, including immune-cell-class effects, meniscal structural-state effects, localization and ordering summaries, and cross-signature concordance.

Table S11	Primary bulk tissue-deconvolution and immune-PC1-adjusted sensitivity analyses in GSE117525 skeletal muscle and GSE55235 synovium, including MCP-counter results, collinearity diagnostics, directional-signature attenuation, gene-level robustness and adjusted cross-tissue concordance.

Source Data legends

File	Description
Source Data Fig. 1	Bulk DEG and sensitivity-analysis source data for GSE117525 and GSE55235.
Source Data Fig. 2	Shared genes, overlap statistics, enrichment and PPI/Hub-15 source data.
Source Data Fig. 3	GSE157007 quality-controlled immune source data, donor-by-cell-class pseudobulk module and gene-level results, donor coverage, composition and state-ordering summaries.
Source Data Fig. 4	GSE220243 meniscal structural-cell source data, official regional-sample-to-donor mapping, donor-corrected state fractions and state-specific Hub-15 results, threshold sensitivity and ordering/module-dynamics summaries.
Source Data Fig. 5	CellChat source data and receiver/pathway/LR summaries together with donor-level expression-proxy values, effect-size statistics, bootstrap confidence intervals, BH-adjusted P values, donor mapping and coverage audits.

Supplementary figure legends

Supplementary Fig. S1. Bulk DEG quality control and GSE117525 sensitivity analysis.

Supplementary Fig. S2. Enrichment-scope clarification and PPI/Hub-15 robustness.

Supplementary Fig. S3. Peripheral immune atlas, donor-aware Hub-15 pseudobulk analysis and composition. (a) Peripheral immune/hematopoietic atlas of 90,185 major-class-annotated cells from 14 GSE157007 donors. (b) Donor-group context within the shared atlas, with all annotated cells shown in grey and Young (3 donors; 23,822 cells), Healthy older (6 donors; 47,444 cells) or Frail cells (5 donors; 18,919 cells) highlighted in the corresponding facet. (c) Canonical-marker support for the ten displayed broad cell-class annotations; point area denotes the percentage of expressing cells and color denotes gene-wise scaled mean expression. (d) Cell-class localization of the 15 PPI-derived Hub-15 genes using the same encodings. (e) Frail versus Healthy older donor-by-cell-class pseudobulk module effects for the six evaluable classes. Filled points indicate module-level fry BH FDR < 0.05 and open points indicate BH FDR $>= 0.05$; horizontal lines show 2,000-replicate bootstrap 95% confidence intervals for the descriptive standardized pseudobulk-score difference. The six non-evaluable classes are omitted from the effect rows and listed below the panel. (f) Donor-level major-cell-class composition, with each bar normalized within donor and cord-blood donors excluded. (g) Gene-level edgeR log2 fold changes for detected Hub-15 genes in the six evaluable classes; asterisks indicate gene-level BH FDR < 0.05 and light-grey tiles indicate no finite estimate.

Supplementary Fig. S4. Meniscal structural-state annotation, composition and Hub-15 analyses. (a) Disease context on the shared meniscal structural atlas; grey points denote the complete atlas and each facet highlights one disease group. (b) Regional-sample structure using anonymous group-local identifiers, with original identifiers retained in the mapping table; the eight Normal samples represent seven donors because two regions derive from Normal Donor1. (c) Representative structural-state marker support; color is scaled within each gene and point area denotes detectable expression. (d) Sample-level structural-state composition across the 14 regional samples; fractions are descriptive and no donor-level abundance inference is implied. (e) Sample/state fractions of cells above the global structural-atlas top-5% Hub-15 threshold, shown descriptively by disease group and state. (f) OA-Normal donor-median Hub-15 score differences after pooling the two Normal Donor1 regions; horizontal lines show 2,000-replicate bootstrap 95% confidence intervals and right-side values show BH FDR across six states. None of the six states reached BH FDR < 0.05. (g) Precomputed structural state-ordering score by annotated state. (h) Group-specific descriptive LOESS trends for Hub-15, inflammatory/stress, catabolic and fibrochondrocyte/remodeling module scores along the structural ordering; module-specific y-axis scales are used, and the ordering is not interpreted as temporal lineage progression.

Supplementary Fig. S5. Myeloid-to-structural communication networks and ligand-receptor summaries. (a,b) Absolute myeloid-to-structural communication networks in Normal and OA groups using identical probability scales. (c) Retained cell counts by communication group, documenting the cellular input to each inferred network; these counts are technical quality-control summaries

and not donor-level abundance comparisons. (d) CellChat ligand-receptor/database preflight quality control; labels report raw values and color is scaled within each metric row. (e) Top pathway-level OA-Normal communication-potential shifts. (f) Top ligand-receptor pairs, with point area denoting maximum absolute inferred probability and color denoting the OA-Normal difference.

Terminology and variable-name mapping

Key code-to-text mapping

Code variable	Main-text term	Meaning
PPIHub15_Score1	Hub-15 module score	Seurat AddModuleScore output for the PPI-derived 15-gene hub inflammatory/migration module.
SharedInflam_Z_v4	Hub-15 module z-score	Standardized Hub-15 module score used for structural-cell localization; the main figure uses the top 5% threshold and Table S6D provides a top 10% sensitivity output.
F2Shared16_SensitivityScore1	original shared-16 sensitivity score	Sensitivity-only score for the 16 original shared DEGs.
analysis_sample_id	regional sample identifier	GSE220243 regional-sample identifier; GSM6797160 and GSM6797161 derive from the same Normal Donor1 and are pooled into analysis_unit_id = Normal_Donor1 for donor-level inference.
delta_total_prob_OA_minus_Normal	OA–Normal difference in CellChat-inferred communication probability	Difference in inferred communication potential between OA and Normal.
expression_proxy	donor-level ligand × receptor expression proxy	Expression-level plausibility proxy calculated at independent-donor level after pooling repeated regions within donor. Mean linearized myeloid ligand expression is multiplied by target-state receptor expression; the proxy does not establish secretion, binding or causality.
pseudobulk_id	donor-by-cell-class pseudobulk identifier	Identifier formed from donor and mapped major immune-cell class after raw-count aggregation in GSE157007.
fry_BH_FDR	Hub-15 module BH FDR	BH-adjusted P value from the limma-voom fry self-contained Hub-15 gene-set test across evaluable immune-cell classes.
analysis_unit_id	independent donor identifier	Donor-level GSE220243 analysis unit after pooling repeated regional samples from Normal Donor1.
median_Hub15_score	donor-state Hub-15 median	Median continuous Hub-15 score within one donor and structural state, used for Supplementary Fig. 4f inference.
immune_PC1_MCP_counter	MCP-counter immune PC1	First principal component of standardized MCP-counter immune-lineage scores, used only as a bulk immune-adjustment sensitivity covariate.
immune_PC1_xCell	xCell immune PC1	First principal component of standardized xCell immune-enrichment scores; treated as secondary in GSE55235 because of high collinearity with OA status.
directional_score	directionally oriented shared-signature score	Average standardized expression after multiplying each gene by an externally defined disease-direction coefficient to prevent positive and negative signature components from cancelling.
attenuation_percent	immune-adjustment effect attenuation	Percentage reduction in the absolute group-effect magnitude after adjustment for the MCP-counter immune PC1.
